# Investigation of the therapeutic role of native plant compounds against colorectal cancer based on system biology and virtual screening

**DOI:** 10.1038/s41598-023-38134-5

**Published:** 2023-07-15

**Authors:** Abbas Alibakhshi, Rahim Malekzadeh, Sayedeh Azimeh Hosseini, Hajar Yaghoobi

**Affiliations:** 1 Molecular Medicine Research Center, Hamadan University of Medical Sciences, Hamadan, Iran; 2 Department of Medical Biotechnology, School of Advanced Technology, Shahrekord University of Medical Sciences, Shahrekord, Iran; 3 Clinical Biochemistry Research Center, Basic Health Sciences Institute, Shahrekord University of Medical Sciences, Shahrekord, Iran

**Keywords:** Biophysics, Cancer, Computational biology and bioinformatics, Drug discovery, Plant sciences, Systems biology

## Abstract

This study investigated the anticancer effects of compounds extracted from native plants on colon cancer following drug–target-network analysis and molecular docking. Based on the ChEBI database, compounds were identified in medicinal plants and weeds in the Chaharmahal and Bakhtiari provinces of Iran. A drug–target network was constructed based on candidate colon cancer protein targets and selective compounds. Network pharmacology analysis was conducted against the identified compounds and subjected to molecular docking studies. Based on molecular dynamics simulations, the most efficient compounds were evaluated for their anticancer effects. Our study suggests that TREM1, MAPK1, MAPK8, CTSB, MIF, and DPP4 proteins may be targeted by compounds in medicinal plants for their anti-cancer effects. Multiorthoquinone, Liquiritin, Isoliquiritin, Hispaglabridin A, Gibberellin A98, Cyclomulberrin, Cyclomorusin A, and Cudraflavone B are effective anticancer compounds found in targeted medicinal plants and play an important role in the regulation of important pathways in colon cancer. Compounds that inhibit MIF, CTSB, and MAPK8-16 appear to be more effective. Additional in vitro and in vivo experiments will be helpful in validating and optimizing the findings of this study.

## Introduction

Colorectal cancer (CRC) is a common malignant tumor of the gastrointestinal tract with vague early and late symptoms of anemia, weight loss, and other systemic symptoms^1^. In addition to conventional surgical intervention, other treatments are used, including the development of chemotherapy agents, finding new drug carriers, and combination therapy. Due to the side effects of some of these treatments, many studies have been conducted using natural compounds in cancer therapy^2,3^. The identification of natural compounds in herbal medicines that can be used as anti-cancer drugs has significantly reduced the mortality of cancer patients. In addition, natural compounds derived from medicinal plants have excellent anticancer effects as well as unique advantages such as low toxicity, fewer side effects, and lower cost. Several studies have focused on the therapeutic effects of herbal medicines on metastatic cancers. For example, cancer treatment using berberine, curcumin, resveratrol, and other bioactive compounds extracted from medicinal plants has been shown to target different factors that regulate tumor growth and metastasis. In contrast, the malignancy of cancer cells is typically caused by a combination of cellular and molecular factors^4,5^. Therefore, it can be claimed that choosing the proper intracellular factor or pathway as a therapeutic target is the first step in the therapy of such disorders. Identifying the active compounds of medicinal plants that influence various targets involved in the development of cancer cells can be considered an important step in cancer treatment.

Although routine studies based on natural substances, effective substances and their targets have already been selected based on experiences or impressions from previous studies, in such studies, the possibility of interaction between the effective substance and the target based on their physical structures and pharmacokinetic properties is not considered, and the possibility of finding several new potential substances and introducing unknown or less investigated targets is lost. Therefore, one way can be the rigid analysis of previous studies using interpretation of biological data and the examination of molecules in terms of their physicochemical structures for strong interaction with their target, thereby inhibiting its activity, so that it can finally introduce several molecules and lead to a large reduction in the probability of trial and error. Investigating biological interaction networks, including the drug–target interaction network, using data mining and analyzing and simulating their interactions with the help of computer and bioinformatic methods can be very helpful in introducing potential molecules with hidden roles and finally using them in laboratory methods. High-throughput data such as genomic and proteomic data, have contribued significantly to mechanism-based drug discovery. In addition to accelerating drug target identification and screening, bioinformatics analysis also facilitates the identification of side effects and drug resistance. Laboratory investigations of molecular structures, as well as the development of molecular modeling and simulations alongside large molecular databases, have paved the way for more realistic investigations of molecular connections and more informative virtual screening^6^.

The purpose of this study was to use compounds identified from native plants to investigate their possible therapeutic effects on colon cancer using one or more molecular targets. This interaction has been investigated based on pharmacological approaches by modeling drug–target (DT) and drug–target-disease (DTD) networks and analyzing them using molecular docking and MD simulation approaches. Estimating this possible correlation based on the molecular targets involved in different pathways of colon cancer could introduce anticancer compounds with low toxicity and side effects that simultaneously act against different pathways of carcinogenesis, including metastasis, proliferation, and angiogenesis.

## Methods

### Preparation of compounds

All known medicinal plants and weeds in the Chaharmahal and Bakhtiari provinces of Iran were extracted from the flora book of this province and literature review. The names of the plants were searched in the Chemical Entities of Biological Interest (ChEBI) database for potential compounds^7^. ChEBI is a freely available database and the ontology of molecular entities focuses on small chemical compounds. Among all the compounds in this database, the plant compounds identified in the study were downloaded in the SDF format (structural data file). After removing minor and duplicate compounds, the final compounds were selected.

### Preparation of targets

The PharmMapper website (http://www.lilab-ecust.cn/pharmmapper/) was used to identify the potential target candidates for these compounds^8^. This web server finds the best mapping poses of the uploaded molecules against all targets in several web-accessible databases. The target set option was set to human protein targets, and the maximum number of reserved matched targets was set to 300. After eliminating duplicates, desired targets were selected for further evaluation. Colorectal cancer-related targets were identified using the NCBI database (https://www.ncbi.nlm.nih.gov/gene) and matched to the potential targets of the selected compounds. Common targets of the two groups were selected for further investigation.

A drug–target (DT) network was constructed using Cytoscape v3.6.0 software based on candidate protein targets and selective compounds. To provide a network diagram, the targets are represented by circular nodes and compounds by triangle-shaped nodes. The NetworkAnalyzer plugin^9^ was used to analyze the quantitative properties of the undirected network, in which parameters such as degree and betweenness centrality were estimated, and the nodes for each target and compound with the highest degree and betweenness centrality score were selected^10^.

### Protein and ligand preparation and molecular docking study

Targeted proteins were selected from the network analysis, and their structures were retrieved in PDB format from the Protein Data Bank (http://www.rcsb.org/PDB) and converted to pdbqt using AutoDock software^11^. In addition, the selected compound structures in sdf format were converted into mol2 and pdbqt formats using OpenBabel and Raccoon software, respectively^12^. Vina software hosted in PyRx, a virtual screening software^13^, was used for docking, and PyMol and DiscoveryStudio were used to analyze docked complexes.

### Pharmacokinetic and ADME study

Compounds that showed interaction energy above − 7.5 kcal/mol against their targets were checked by the SwissADME website^14^ in terms of physicochemical descriptors, as well as to predict ADME parameters (absorption, distribution, metabolism, and excretion), pharmacokinetic properties, and drug-like nature using the Lipinski rule.

### Molecular dynamic simulation

United atom molecular dynamics (MD) simulations were performed using the GROMACS 2019.6 software package and the G43a1 force field in combination with the SPC water model for all protein and ligand–protein complexes. The system neutralization and physiological ionic strength of 0.14 M were achieved by adding suitable amounts of Na^+^ and Cl^−^ ions^15^.

First, energy minimization for the relaxation of internal constraints was performed using the steepest descent method until the system converged. Equilibration in the NVT and NPT ensembles was then performed under positional restraints for 500 and 1000 ps, respectively. Finally, an MD production run was performed for 100 ns with a 2 fs time step for all proteins and ligand–protein complexes.

The Ewald particle mesh^16^ was used for long-range electrostatic forces, and the lengths of all bonds were constrained using the LINCS algorithm^17^. The temperature was set to 310 K using a V-rescale thermostat^18^ and the pressure was controlled at 1.0 atm using a Parrinello–Rahman barostat^19^.

### MM/PBSA binding free energy calculation

The molecular mechanics Poisson–Boltzmann surface area (MM/PBSA) approach^20^ was applied to calculate the binding free energy of protein–ligand interactions using the last 50 ns of each simulation trajectory.

The total binding energy of the system was calculated using the following equation:

$$\Delta {\text{G}}_{{{\mathrm{Binding}}}} = \Delta {\text{G}}_{{{\mathrm{Complex}}}} {-}\Delta {\text{G}}_{{{\mathrm{Ligand}}}} {-}\Delta {\text{G}}_{{{\mathrm{Receptor}}}}$$

### Principal component analysis

We performed principal component analysis (PCA) of the α-carbon atoms in the 100 ns trajectories to further explore the dynamic properties of the investigated structures. PCA was obtained as the eigenvectors of a covariance matrix consisting of α-carbon displacements that reflects the overall conformational motions and flexibility of the systems during the simulations.

The first two principal components (PC1 and PC2) that dominate protein conformational fluctuations were used to analyze the proteins and protein–ligand complexes. The eigenvalue corresponding to each eigenvector represents the energy contribution of that particular part of the motion. Eigenvectors and eigenvalues were generated using the g_covar Gromacs utility by calculating and diagonalizing the covariance matrix. Eigenvector analysis was performed using the g_anaeig tool.

### Ethical approval

This article does not contain any studies involving human participants or animals performed by any of the authors.

## Results

### Libraries preparation

A total of 265 medicinal plants were selected and introduced into the ChEBI database to extract the effective compounds. After removing duplicates, 87 potential compounds were obtained from the 265 native plant species. The PharmMapper website has introduced 423 potential target candidates for these compounds. In addition, the NCBI database has introduced 3312 colorectal cancer-related targets. Finally, 34 human protein molecules were selected, which could be targets of these compounds and play a role in colorectal cancer.

Of these compounds, 7 did not have common targets in their top 30 targets. The names and 2D structures of both the groups of compounds used in the screening and their targets are listed in Supplementary Tables S1–S3.

### DT network construction and analysis

**Figure 1:**
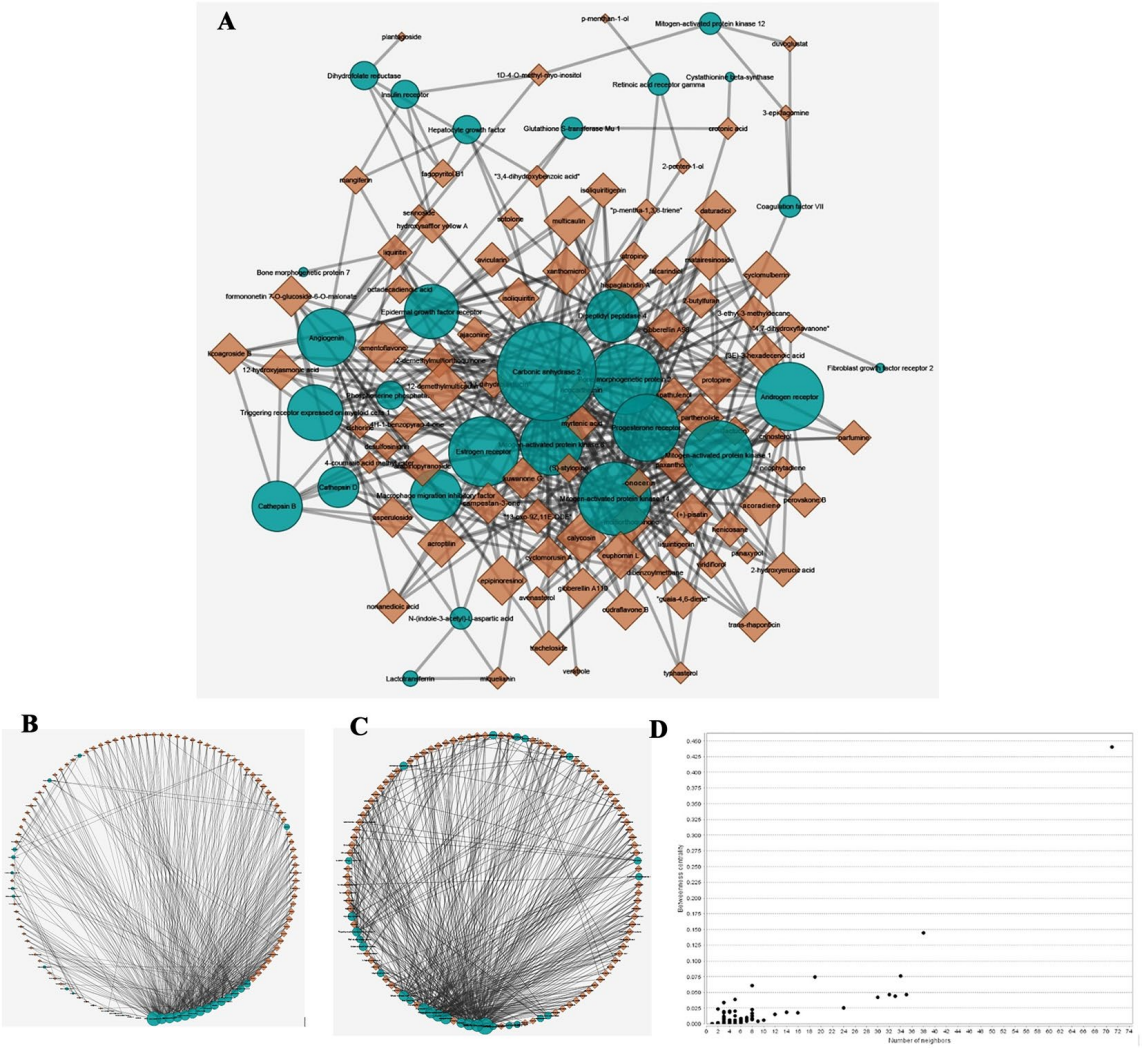
(**A**) The bipartite DT network. (**B**) The circle layout of DT network based on degree. (**C**) The circle layout of DT network based on betweenness centrality. (**D**) The betweenness centrality of nods. The green circular nodes represent the targets, and the brown triangular nodes represent the chemical compounds. The larger the size of the node, the greater the number of nodes and associations.

A DT network with 87 compounds and 34 targets was constructed consisting of 114 nodes and 416 degrees. The NetworkAnalyser results calculate parameters such as the number of degrees, betweenness, and edge betweenness centrality values, which are used to select the best molecular targets and potential chemical compounds (Fig. 1). Figure 1A shows a bipartite network configured with node sizes based on the number of degrees; the larger the node size, the greater is the number of associations. Based on the network analysis, the compounds showed degrees ranging from 1 to 8. To screen the best compounds as ligands for molecular docking analysis against targets, compounds with six or more nodes were selected. Therefore, 31 compounds listed in Table 1 were selected for further study. Carbonic anhydrase 2, with a score of 0.44, showed the highest centrality among the targets, followed by mitogen-activated protein kinase 14 (0.14), estrogen receptor (0.07), and angiogenin (0.07). The remaining molecules had a score of less than 0.05. Moreover, Carbonic anhydrase 2 (71), mitogen-activated protein kinase 14 (38), bone morphogenetic protein 2 (35), and estrogen receptor (34) showed the most connections, based on the number of degrees associated with the nodes.

**Table 1:** The compounds that were selected for molecular docking based on the number of degrees.

Compound name	Degree	Compound name	Degree
12-Demethylmulticaulin	8	Formononetin 7-*O*-glucoside-6-*O*-malonate	6
Amentoflavone	8	Gibberellin A110	6
Calycosin	8	Hispaglabridin A	6
Epipinoresinol	8	Isoliquiritin	6
Multicaulin	8	Licoagroside B	6
Protopine	8	Matairesinoside	6
Acroptilin	7	Cyclomorusin A	6
Cyclomulberrin	7	Daturadiol	6
Parthenolide	7	Gibberellin A98	6
Xanthomicrol	7	“Guaia-4,6-diene”	6
Euphornin L	7	Multiorthoquinone	6
Arabinopyranoside	6	β-Acoradiene	6
(+)-Pisatin	6	12-Hydroxyjasmonic acid	6
(3E)-3-Hexadecenoic acid	6	Asperuloside	6
5α ± -Campestan-3-one	6	Kuwanone G	6
Cudraflavone B	6		

Finally, the compounds showed a number of degrees ranging from 1 to 8. To screen the best compounds as ligands for the molecular docking step against the targets, all compounds with six or more nodes were selected, in which there were 31 compounds with six, seven, or eight nodes.

### Molecular docking and simulation

**Figure 2:**
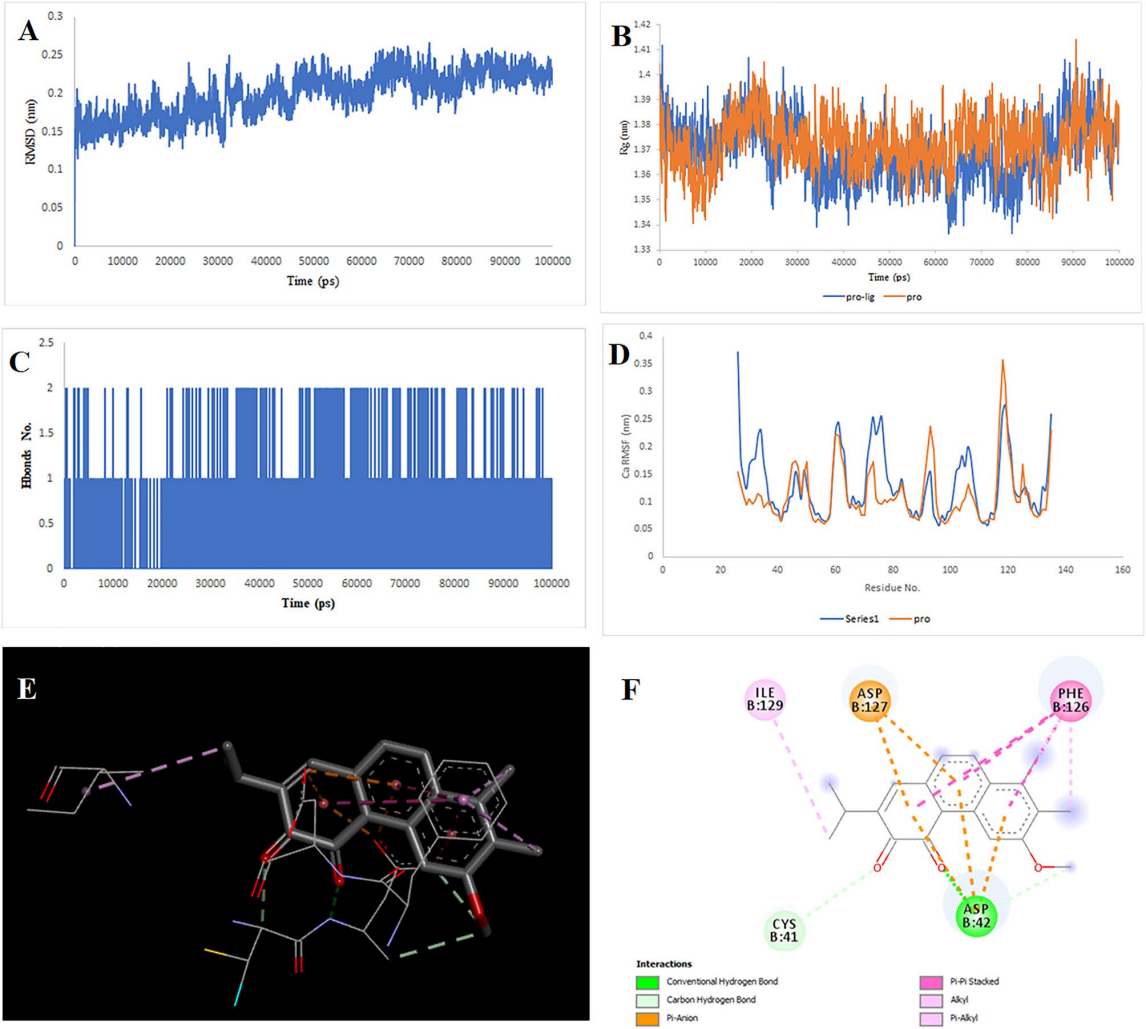
Two-dimensional representations of the Multiorthoquinone against TREM1. (**A**) Root-mean square deviation of the complexes (RMSD). (**B**) Radius of gyration (Rg). (**C**) Hydrogen bond analysis from the simulation system. (**D**) Root-mean-square fluctuation (RMSF). (**E**) The binding conformation of 3D view. (**F**) Binding site interactions of 2D view.

**Figure 3:**
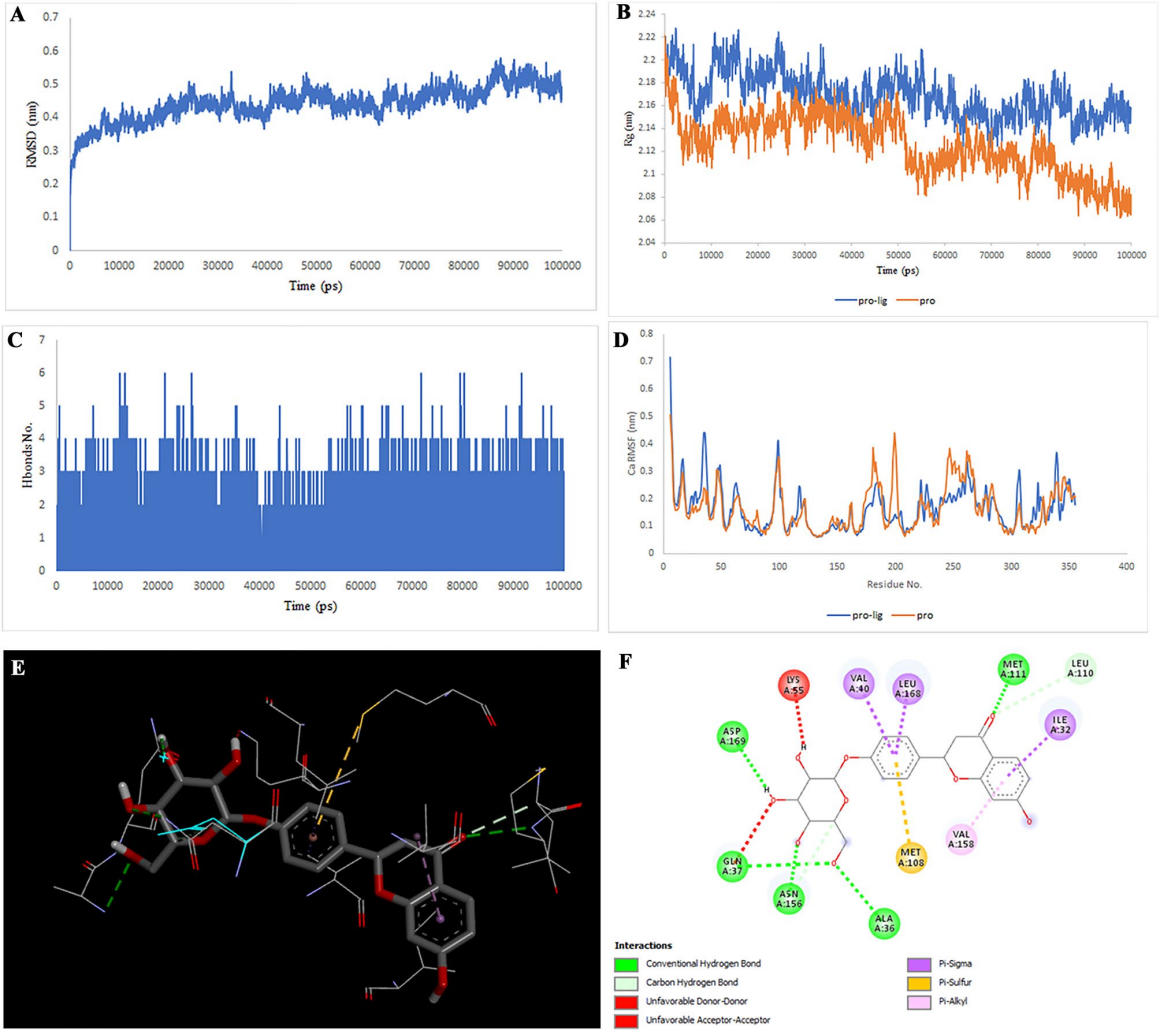
Two-dimensional representations of the Liquiritin against MAPK8. (**A**) Root-mean square deviation of the complexes (RMSD). (**B**) Radius of gyration (Rg). (**C**) Hydrogen bond analysis from the simulation system. (**D**) Root-mean-square fluctuation (RMSF). (**E**) The binding conformation of 3D view. (**F**) Binding site interactions of 2D view.

**Figure 4:**
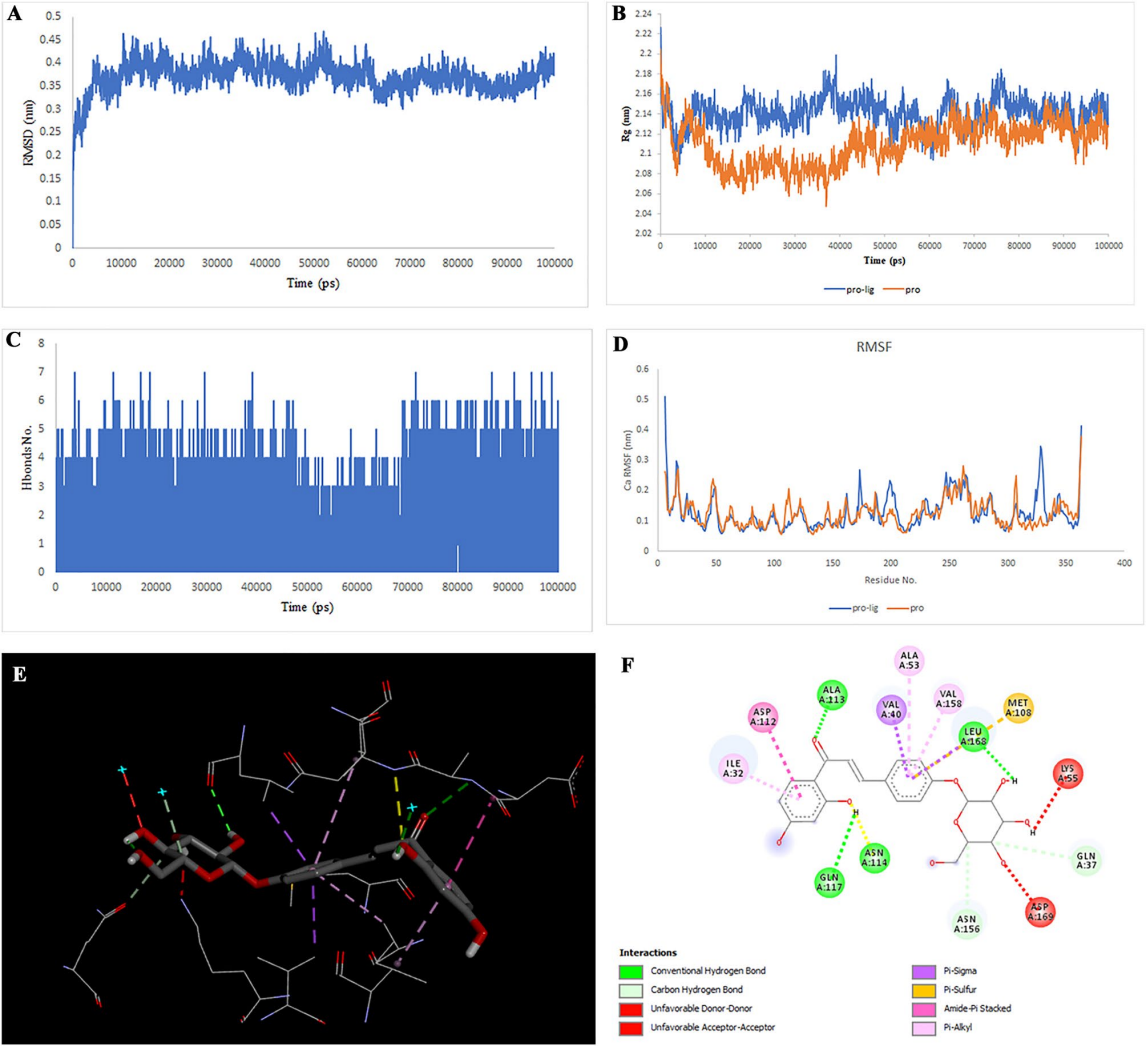
Two-dimensional representations of the Isoliquiritin against MAPK8. (**A**) Root-mean square deviation of the complexes (RMSD). (**B**) Radius of gyration (Rg). (**C**) Hydrogen bond analysis from the simulation system. (**D**) Root-mean-square fluctuation (RMSF). (**E**) The binding conformation of 3D view. (**F**) Binding site interactions of 2D view.

**Figure 5:**
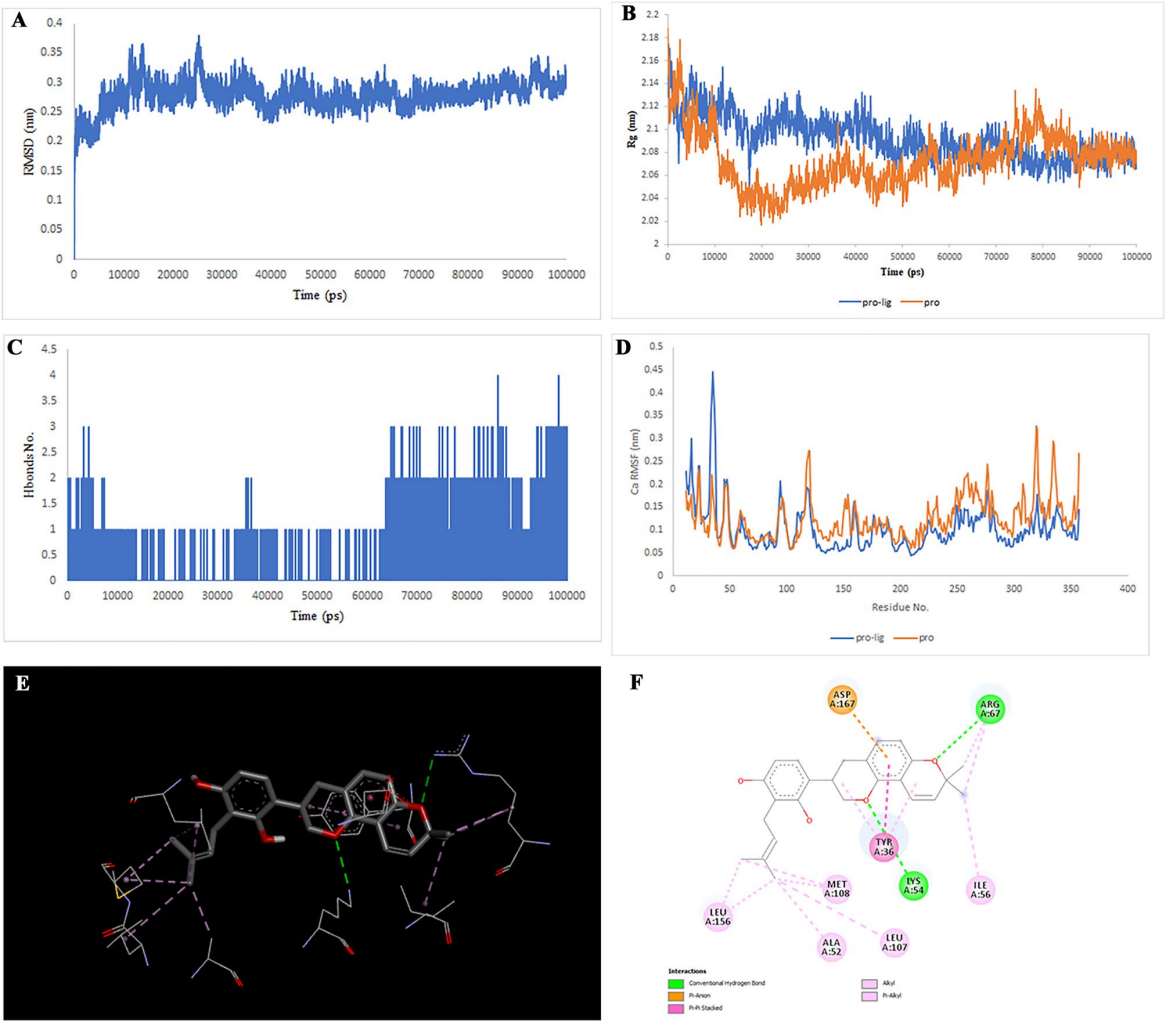
Two-dimensional representations of the Hispaglabridin A against MAPK1. (**A**) Root-mean square deviation of the complexes (RMSD). (**B**) Radius of gyration (Rg). (**C**) Hydrogen bond analysis from the simulation system. (**D**) Root-mean-square fluctuation (RMSF). (**E**) The binding conformation of 3D view. (**F**) Binding site interactions of 2D view.

**Figure 6:**
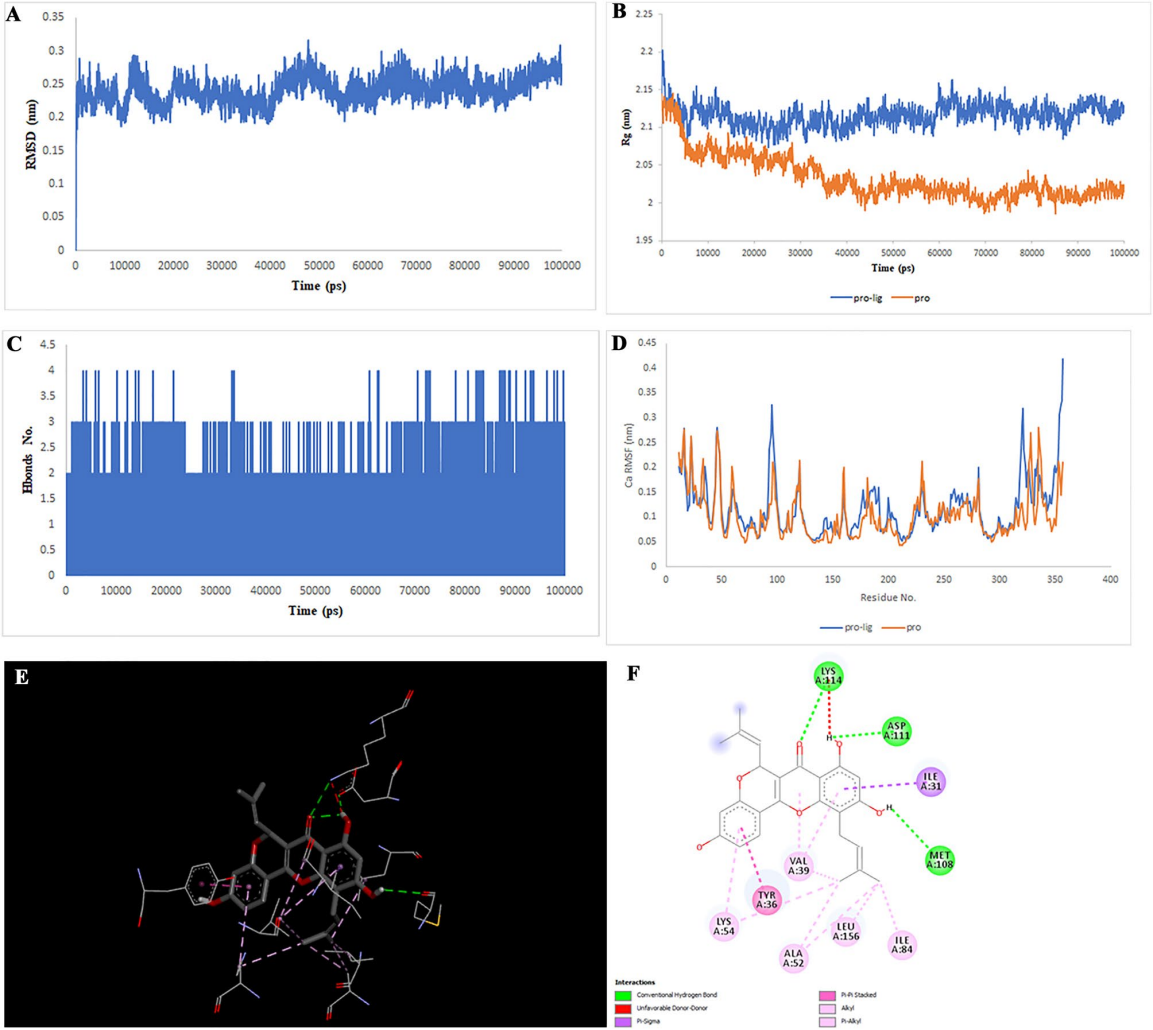
Two-dimensional representations of the Cyclomulberrin against MAPK1. (**A**) Root-mean square deviation of the complexes (RMSD). (**B**) Radius of gyration (Rg). (**C**) Hydrogen bond analysis from the simulation system. (**D**) Root-mean-square fluctuation (RMSF). (**E**) The binding conformation of 3D view. (**F**) Binding site interactions of 2D view.

**Figure 7:**
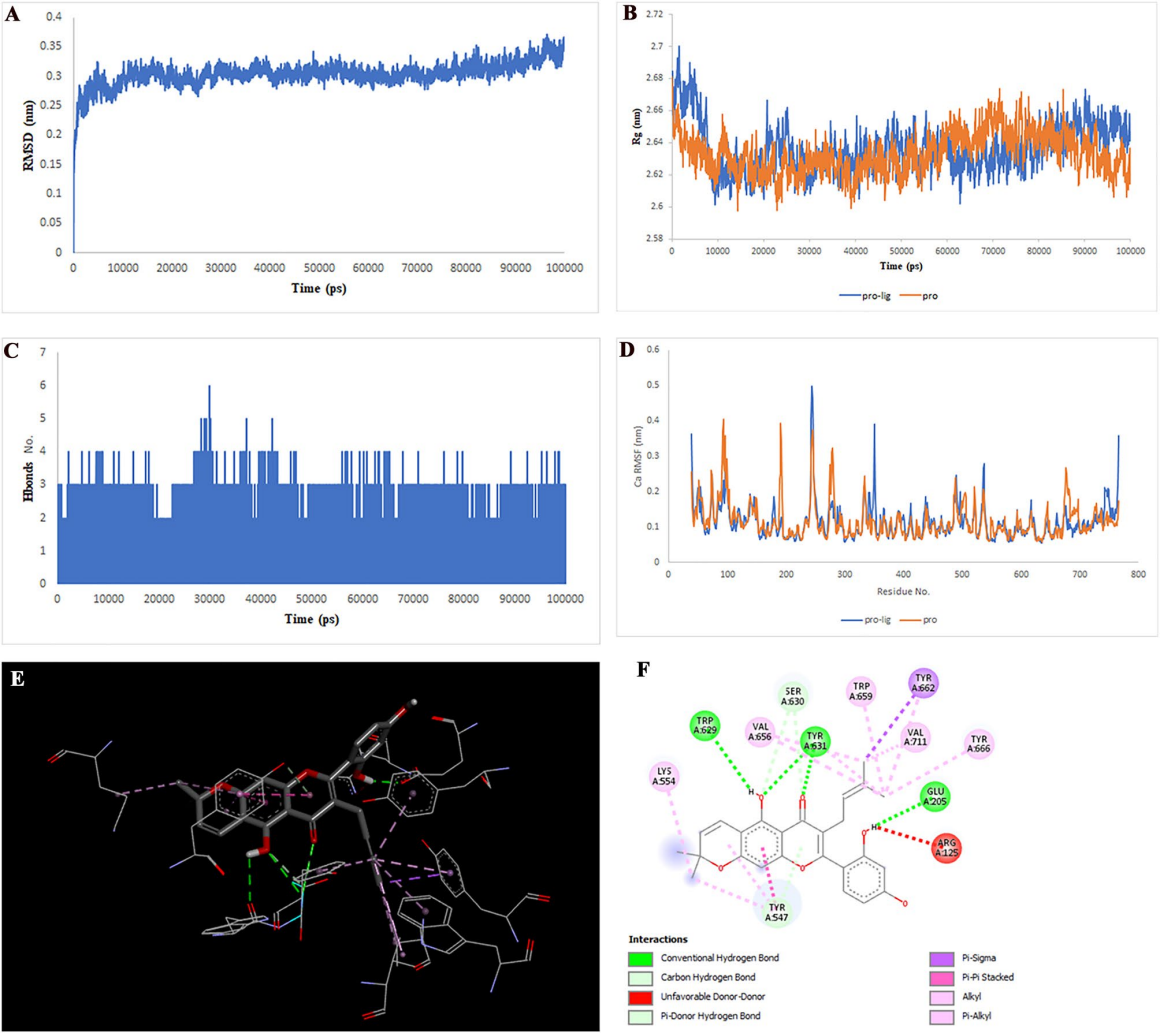
Two-dimensional representations of the Cudraflavone B against DPP4. (**A**) Root-mean square deviation of the complexes (RMSD). (**B**) Radius of gyration (Rg). (**C**) Hydrogen bond analysis from the simulation system. (**D**) Root-mean-square fluctuation (RMSF). (**E**) The binding conformation of 3D view. (**F**) Binding site interactions of 2D view.

**Figure 8:**
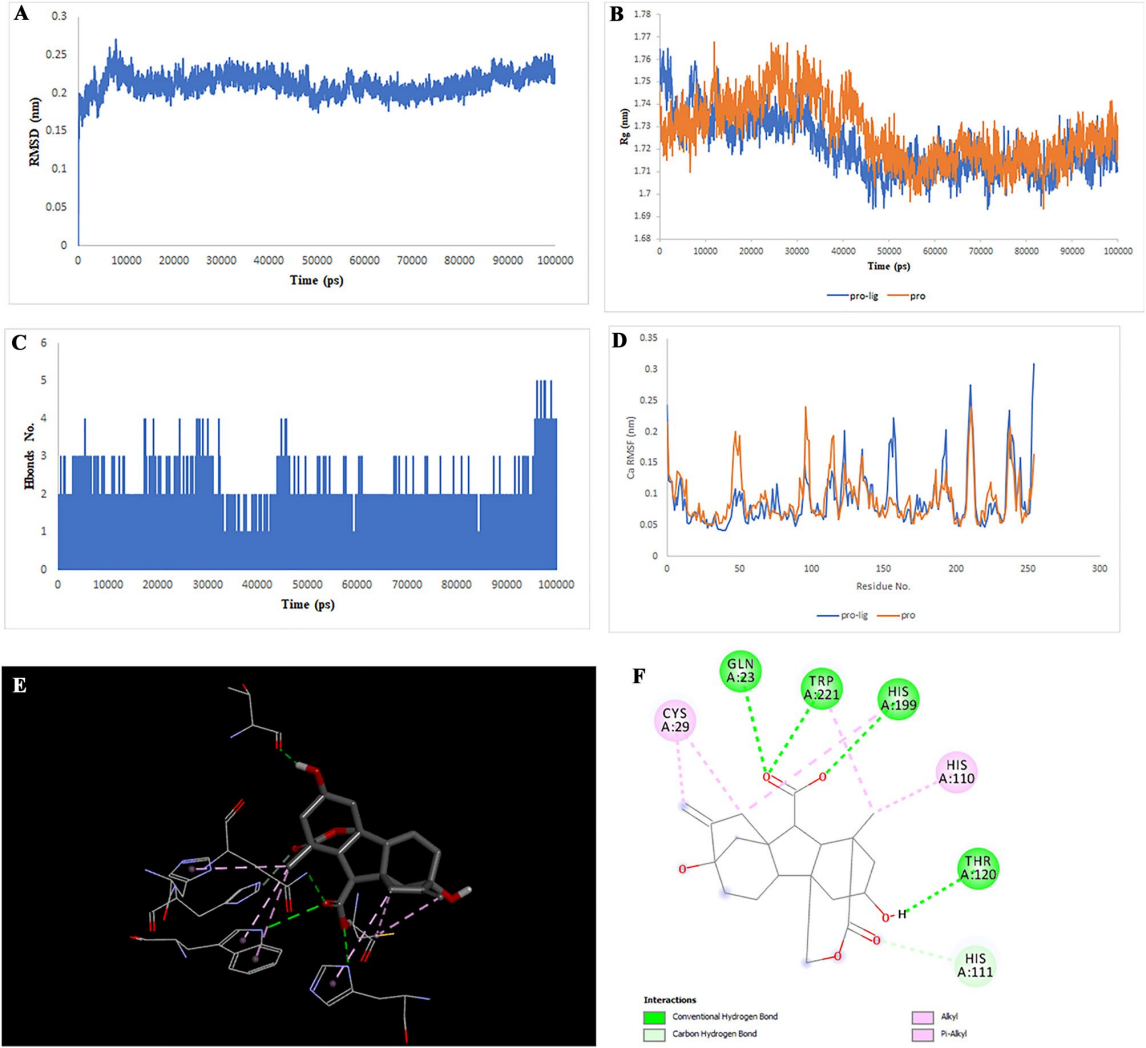
Two-dimensional representations of the Gibberellin A98 against CTSB. (**A**) Root-mean square deviation of the complexes (RMSD). (**B**) Radius of gyration (Rg). (**C**) Hydrogen bond analysis from the simulation system. (**D**) Root-mean-square fluctuation (RMSF). (**E**) The binding conformation of 3D view. (**F**) Binding site interactions of 2D view.

**Figure 9:**
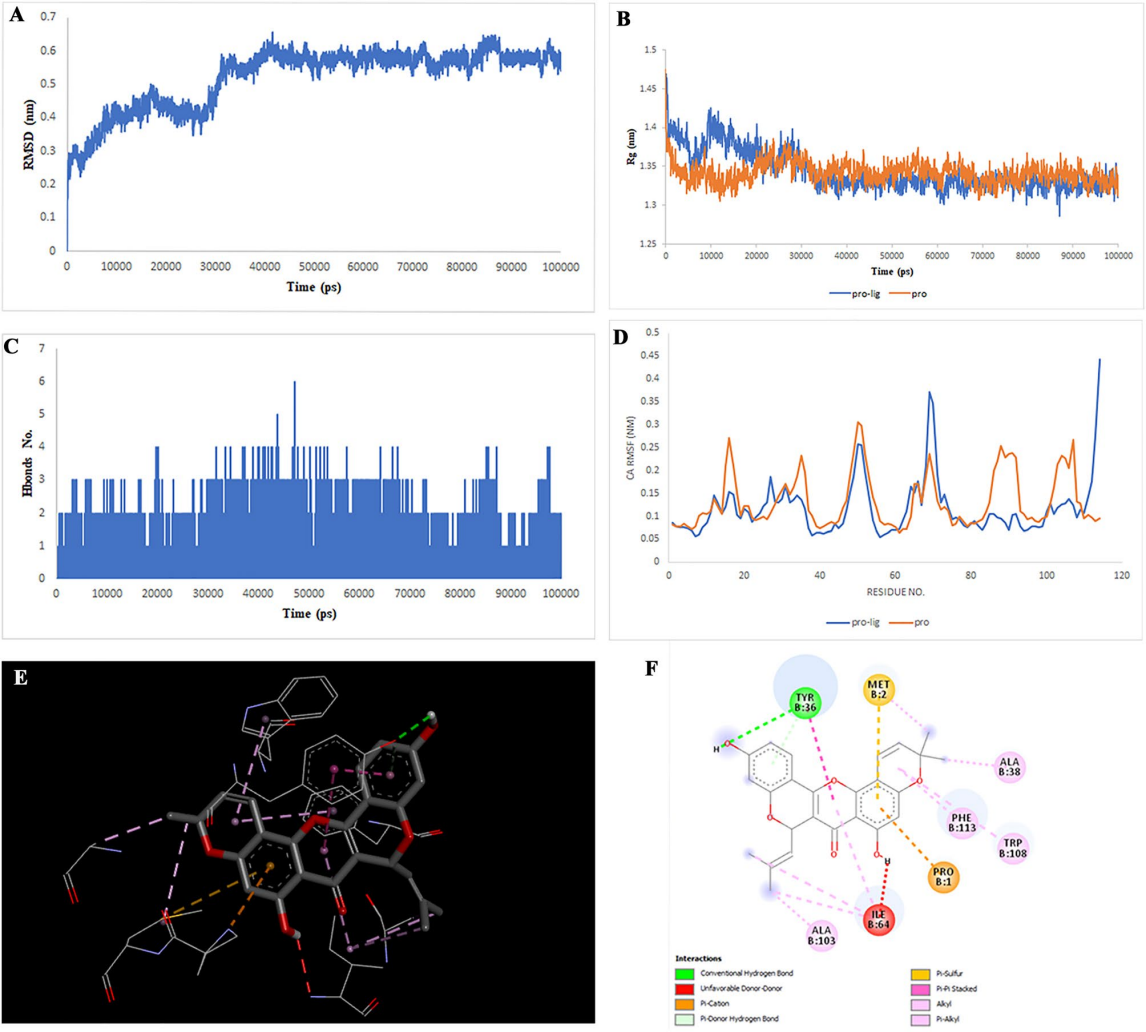
Two-dimensional representations of the Cyclomulberrin against MIF. (**A**) Root-mean square deviation of the complexes (RMSD). (**B**) Radius of gyration (Rg). (**C**) Hydrogen bond analysis from the simulation system. (**D**) Root-mean-square fluctuation (RMSF). (**E**) The binding conformation of 3D view. (**F**) Binding site interactions of 2D view.

After checking using the GEPIA database, the TOP targets that showed increased expression in colorectal cancer were selected. The target structures were downloaded from the PDB database (PDB IDs: 6AY2 for CTSB, 5T4E for DPP4, 6FVE for MIF, 7AUV for MAPK1, 4QTD for MAPK8, and 1SMO for TERM1). The Chimera 1.8.1 software was used for essential protein preparations, including removing water, ATP, ligands, and adding hydrogen charges. Critical ligand-binding sites were considered and docked to 31 compounds using the PyRx software. 31 molecules were selected after analyzing the pharmacokinetic properties and parameters of ADME. The centroid of the binding sites for the targets was calculated as the coordinates of the centroid of the ligand-binding sites using the UniProt database. The docked complexes were analyzed using PyMol and DiscoveryStudio software. Finally, molecules that presented the highest interaction energy against their target (above − 7.5 for TERM1, − 8 for CTSB and MIF, above − 9 for DPP4 and MAPK1 and above − 10 for MAPK8), respectively and had more binding with the amino acids of the binding site in the study with DiscoveryStudio. These findings led to the selection of Multiorthoquinone, Liquiritin, Hispaglabridin A, Isoliquiritin, Gibberellin A98, Cyclomulberrin, Cyclomorusin A, Cudraflavone B for simulation (MD), which produced a more stable complex with a lower energy level than TREM1, MAPK8, MAPK1, MAPK8, CTSB, MAPK1, MIF, and DPP4, respectively. The positions and amino acids involved in the binding are illustrated in Fig. 2, 3, 4, 5, 6, 7, 8 and 9, Supplementary Tables S4–S6. The RMSD score showed slight fluctuations and was approximately 0.2 nm. These findings indicate the stability of the compounds in the target complex. The RMSF values for all protein structures were computed to accurately determine how the binding of the compounds affects flexibility.

Analysis of the liquiritin- and isoliquiritin-associated RMSF plots revealed that Liquiritin and Isoliquiritin flexibility were significantly different in the five regions of 35, 183, 199, 246, and 307 and two regions in 200 and 328 in MAPK8, respectively. In addition, multiorthoquinone flexibility was extremely high in the three regions 34, 76, and 107 in TREM1, which may be attributed to a lack of interaction between the three regions and multiorthoquinone. For Cyclomulberrin and Hispaglabridin A residues 94–320, and 35, 300–350 in MAPK1, the flexibility of amino acids was lower and higher, respectively. The RMSF plot associated with DPP4 indicated high interaction between most regions of DPP4 and Cudraflavone B. Additionally, Cyclomorusin A and Gibberellin A98 flexibility were extremely different in the two regions of 50–70 and 150–170 in the CTSB, and 80–100 region in the MIF, respectively.

RG nature was constant for the individual domains during the entire simulation period associated with Gibberellin A98, Cyclomorusin A, and Cudraflavone B, indicating that the individual domains did not melt or unfold. These compounds did not affect the secondary structures of CTSB, MIF, or DPP4. However, the RG value associated with Liquiritin, Isoliquiritin and Hispaglabridin A, Cyclomulberrin throughout the MD simulation led to unfolding and activation of MAPK8 and MAPK1, respectively.

### Principal component analysis

Supplementary Fig. S1 shows the projection of the trajectories on special eigenvectors (vectors 1 and 2) and time-dependent motions of the components in a particular vibration mode. The overall analysis of the eigenvector plots indicated that most vibrations occurred along eigenvector 1. According to the first two PCs, TREM1 and MIF proteins have almost the same trace values of the covariance matrix for the bound and unbound states with a slight shift. This indicates that the ligand is well equilibrated and stabilized with the protein, as reflected by theleast conformational changes due to reduced collective motions from unbound states. However, in other cases, 2D projection plots of the trajectories revealed that the ligand reduced the conformational diversity during the simulations, leading to a more compact cluster distribution. Sampling of different regions and showing different movement behaviors of protein–ligand complexes compared to unbound proteins points to the binding of ligand effects on the rigidity of the structural conformation, which also affects the function of proteins.

### MM/PBSA binding free energy

The MM/PBSA binding free energy results are shown in Table 2 including the van der Waals energy (kJ/mol), electrostatic energy (kJ/mol), polar solvation energy (kJ/mol), SASA energy (kJ/mol), SAV energy (kJ/mol), WCA energy (kJ/mol), and binding energy (kJ/mol), are shown in Table 2. Compared to liquiritin, isoliquiritin had the lowest binding energy score of 183.04 kJ/mol interacting with MAPK8 and formed a stronger binding. In addition, hypoglabrin A and cyclomulbrin interact with MAPK1 at almost the same binding energy (− 154 kJ/mol). However, the number of hydrogen bonds in the interaction with hypoglabrin A is relatively high. MIF-Cyclomorusin A, with the most negative energy score (− 243.768 kJ/mol), showed the strongest interaction among all docked complexes.

**Table 2:** MM-PBSA results.

Energy Forms (kJ/mol)	TREM1—Multiorthoquinone	MAPK8—Liquiritin	MAPK8—Isoliquiritin	MAPK1—Hispaglabridin A	MAPK1—Cyclomulberrin	CTSB—Gibberellin A98	MIF—Cyclomorusin A	DPP4—Cudraflavone B
Van der Waal	− 158.643 ± 1.833	− 200.289 ± 3.237	− 207.328 ± 3.231	− 228.656 ± 1.11	1.591 ± 1.309	− 176.028 ± 0.805	− 259.193 ± 1.262	− 259.028 ± 1.065
Electrostatic	− 30.848 ± 1.237	− 4.575 ± 0.806	− 33.317 ± 1.116	− 3.96 ± 0.334	1.591 ± 0.907	− 22.762 ± 1.556	− 38.108 ± 0.644	− 25.021 ± 0.6
Polar solvation	49.704 ± 1.332	59.473 ± 1.569	76.214 ± 1.464	99.315 ± 1.622	1.591 ± 1.63	68.8 ± 0.737	72.135 ± 1.018	84.784 ± 0.694
SASA	− 12.637 ± 0.113	− 18.136 ± 0.313	− 18.967 ± 0.307	− 20.1 ± 0.1	1.591 ± 0.092	− 13.791 ± 0.056	− 18.568 ± 0.089	− 19.461 ± 0.071
SAV	0	0	0	0	0	0	0	0
WCA	0	0	0	0	0	0	0	0
Binding	− 152.141 ± 2.088	− 163.657 ± 3.341	− 183.044 ± 3.251	− 153.478 ± 1.591	± 1.591 ± 1.678	− 143.916 ± 1.308	− 243.768 ± 1.184	− 218.717 ± 1.12

*SASA* solvent accessible surface area, *SAV* solvent accessible volume, *WCA* weeks-chandler-andersen potential.

## Discussion

Recently, phytoscience researchers have described a wide range of bioactive chemicals with various biological effects on plants. This issue has led to the allocation of pharmaceutical resources for the investigation and introduction of novel drugs from herbal sources. In this regard, identifying natural bioactive components and therapeutically active chemicals through the screening of plant extracts is the first step in the development of these drugs. A network pharmacology analysis, represented by the bipartite DT network, was performed to evaluate the potential interactions between the chemical compounds of medicinal plants and proteins identified in colon cancer. The results of the investigation of the target genes related to the signaling pathways of colon cancer and the names of the plant compounds are presented in Supplementary Tables S1 and S2.

Findings identified the genes MAPK1, MAPK8, TREAM1, CTSB, MIF, and DPP4 interacted most effectively with the compounds. Liquiritin and Isoliquiritin from Glycyrrhiza glabra effectively interact with MAPK8. In addition, Cyclomulberrin and Hispaglabridin A from Morus species and Glycyrrhiza glabra effectively inhibited MAPK1. Previously, Hispaglabridin A isoflavonoid isolated from Glycyrrhiza glabra was introduced as a potential agent for the development of an effective drug for colon cancer^21,22^. Many previous studies have provided evidence that Liquiritin and Isoliquiritin have anticarcinogenic activity in various types of cancer cells, especially colon cancer cells, by regulating different pathways, including PI3K/AKT, P53/P21, and miR-671/HOXB3 signaling. These compounds show anticancer activity, such as inhibition of tumor growth and cell proliferation and promotion of chemosensitivity and cell apoptosis^23–29^. MAPKs are one of the most important factors that regulate cell proliferation in cancer by regulating growth factor receptors. Disrupting their regulation could be an approach in cancer therapy. This study confirmed the inhibitory effects of these compounds on cell proliferation through the introduction of a new target.

C-cudraflavone derivatives showed anticancer activity in human colorectal, melanoma, and oral cancer by regulating the MAPK, SIRT1, NF-κ B, and PI3K/AKT pathways^30–32^. The results of this study showed that the B-cudraflavone derivative also interacted with DPP4. Dipeptidyl peptidase 4 (DPP4) is a cell surface protein that acts as a tumor suppressor or activator depending on its expression. An in vitro analysis is required to investigate the function of DPP4 and the effect of cudraflavone on its function in colon cancer.

Autophagy is one of the strategies used by cancer cells to escape cell death. Increased CTSB levels are required to complete autophagy, and studies have shown that treatment with compounds that induce ERK phosphorylation reduces CTSB levels and blocks autophagy. Several studies have confirmed the oncogenic role of this protein in cell proliferation, invasion, metastasis, and drug resistance. Chemotherapy based on CTSB-depleting compounds suppresses autophagy rescue in cancer cells, ultimately leading to increased antitumor effects^33–35^. This study confirmed that cyclomulberrin induced CTSB folding and inhibited CTSB function. ERK-activating compounds, such as Liquiritin and Isoliquiritin, can be used in combination with cyclomulberrin to study the inhibition of drug resistance and proliferation in solid tumors, especially in colon cancer.

Multi-orthoquinones, Gibberellin A98, and Cyclomorusin A interact with TREM1, MAPK1, and MIF signaling pathways and have selective anti- or tumor-inducing activities. These results provided new insights into the mechanisms of action of these compounds. However, further studies are required to determine its exact effects.

## Conclusion

Network pharmacology analysis revealed the key pathways involved in the anti-cancer activities induced by natural compounds found in medicinal plants. Eight pathways were obtained from the drug–target network analysis (MAPK1, MAPK8, TREM1, CTSB, MIF, and DPP4), and molecular docking studies of these pathways revealed that the identified chemical compounds had strong binding affinities with these pathway components. The current study revealed that Multiorthoquinone, Liquiritin, Isoliquiritin, Hispaglabridin A, Gibberellin A98, Cyclomulberrin, Cyclomorusin A, and Cudraflavone B are effective anticancer compounds found in target medicinal plants and play an important role in the regulation of important pathways in colon cancer. Compounds that inhibit MIF, CTSB, and MAPK8-16 appear to be more effective. Additional in vitro and in vivo experiments will be helpful for validating and optimizing the findings of this study.

## Supplementary Information

Supplementary Information.

## Data Availability

The datasets generated and/or analyzed during the current study are available upon request from the corresponding author.
