# Supplementary material for: Investigation of the therapeutic role of native plant compounds against colorectal cancer based on system biology and virtual screening
Source: Sci Rep. 2023 Jul 15;13:11451. doi: 10.1038/s41598-023-38134-5 (PMC10349871; doi:10.1038/s41598-023-38134-5)
Supplement: Supplementary file 1 — Supplementary Information. [file 41598_2023_38134_MOESM1_ESM.docx]

**Supplementary Table S1.** The names of all targets.

|  | Estrogen receptor |
| --- | --- |
|  | Androgen receptor |
|  | Carbonic anhydrase 2 |
|  | Progesterone receptor |
|  | Macrophage migration inhibitory factor |
|  | Phosphoserine phosphatase |
|  | Epidermal growth factor receptor |
|  | Mitogen-activated protein kinase 14 |
|  | Dipeptidyl peptidase 4 |
|  | Angiogenin |
|  | Cystathionine beta-synthase |
|  | Dihydrofolate reductase |
|  | Nuclear receptor subfamily 1 group I member 2 |
|  | Hepatocyte growth factor |
|  | Retinoic acid receptor gamma |
|  | Glutathione S-transferase Mu 1 |
|  | Retinoic acid receptor beta |
|  | Apoptotic protease-activating factor 1 |
|  | Insulin receptor |
|  | Cathepsin B |
|  | Coagulation factor VII |
|  | Mitogen-activated protein kinase 1 |
|  | Bone morphogenetic protein 2 |
|  | Mitogen-activated protein kinase 8 |
|  | Fibroblast growth factor receptor 2 |
|  | Heme oxygenase 1 |
|  | Mitogen-activated protein kinase 12 |
|  | Lactotransferrin |
|  | Adenosyl homocysteinase |
|  | Cathepsin D |
|  | Triggering receptor expressed on myeloid cells 1 |
|  | Catalase |
|  | Glutathione synthetase |
|  | Bone morphogenetic protein 7 |

**Supplementary Table S2.** The names of all compounds.

| (+)-pisatin | (3E)-3-hexadecenoic acid | (S)-stylopine | 11β,13-dihydrolactucin | 12-demethylmulticaulin | 12-demethylmultiorthoquinone | 12-hydroxyjasmonic acid | 13-oxo-9Z,11E-ODE | 1D-4-O-methyl-myo-inositol | 2-butylfuran | 2-hydroxyerucic acid |
| --- | --- | --- | --- | --- | --- | --- | --- | --- | --- | --- |
| 2-penten-1-ol | 3,4-dihydroxybenzoic acid | 3-epi-fagomine | 3-ethyl-3-methyldecane | 4,7-dihydroxyflavanone | 4-coumaric acid methyl ester | 4H-1-benzopyran-4-one | 5α-campestan-3-one | acroptilin | ajaconine | amentoflavone |
| arabinopyranoside | asperuloside | atropine | avenasterol | avicularin | calycosin | cichorine | crinosterol | crotonic acid | cudraflavone B | cyclomorusin A |
| cyclomulberrin | daturadiol | desulfosinigrin | dibenzoylmethane | duvoglustat | epipinoresinol | euphornin L | fagopyritol B1 | falcarindiol | formononetin 7-O-glucoside-6-O-malonate | gibberellin A110 |
| gibberellin A98 | guaia-4,6-diene | henicosane | hispaglabridin A | hydroxysafflor yellow A | isoliquiritigenin | isoliquiritin | kuwanone G | lactucin | licoagroside B | liquiritigenin |
| liquiritin | mangiferin | matairesinoside | miquelianin | multicaulin | multiorthoquinone | myrtenic acid | N-(indole-3-acetyl)-L-aspartic acid | neocarthamin | neophytadiene | nonanedioic acid |
| octadecadienoic acid | p-mentha-1,3,8-triene | p-menthan-1-ol | Panaxynol | parfumine | parthenolide | paxanthonin | Perovskone B | plantagoside | protopine | sennoside |
| sotolone | spathulenol | Tracheloside | trans-rhaponticin | typhasterol | veratrole | viridiflorol | xanthomicrol | α-onocerin | β-acoradiene |  |

**Supplementary Table S3. Names and 2D structures of 31 molecules used in the screening**

| **Compound Name** | **2D structure** | **Compound Name** | **2D structure** |
| --- | --- | --- | --- |
| 12-demethylmulticaulin | 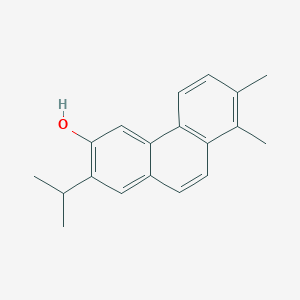 | formononetin 7-O-glucoside-6-O-malonate | 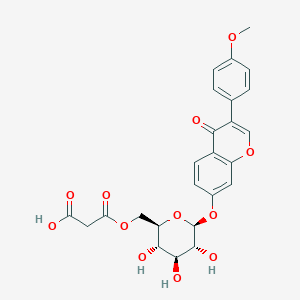 |
| amentoflavone | 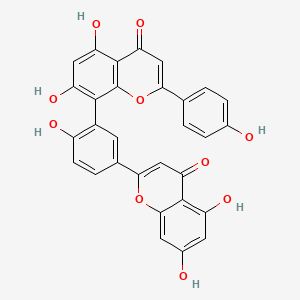 | gibberellin A110 | 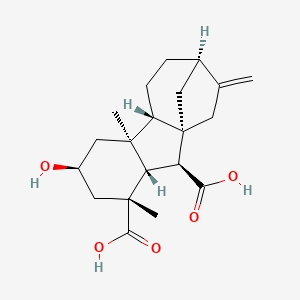 |
| calycosin | 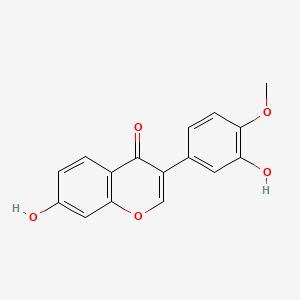 | hispaglabridin A | 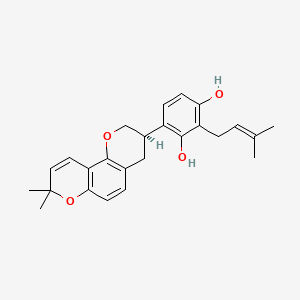 |
| epipinoresinol | 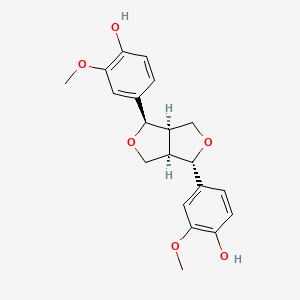 | isoliquiritin | 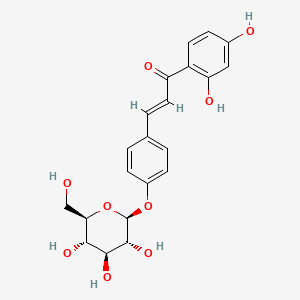 |
| multicaulin | 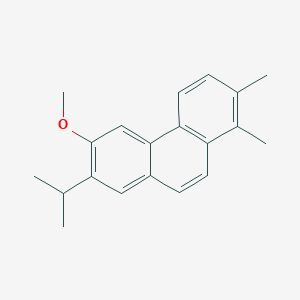 | licoagroside B | 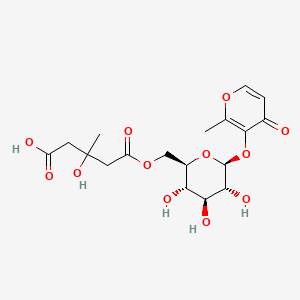 |
| protopine | 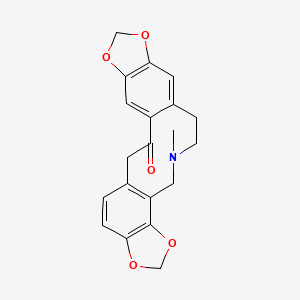 | matairesinoside | 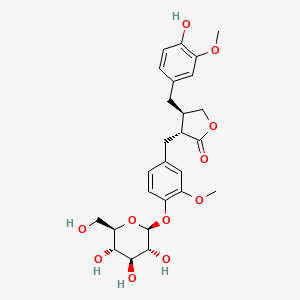 |
| acroptilin | 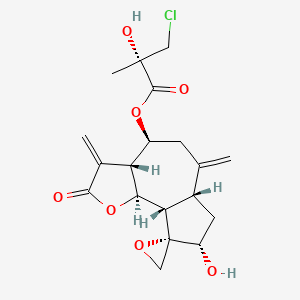 | cyclomorusin A | 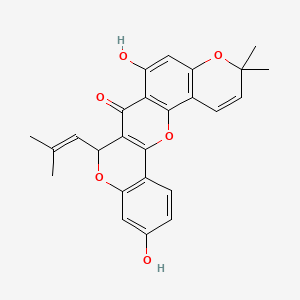 |
| cyclomulberrin | 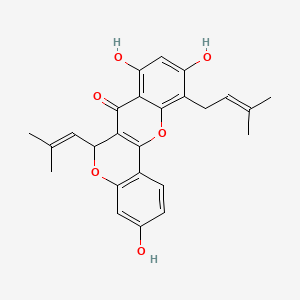 | daturadiol | 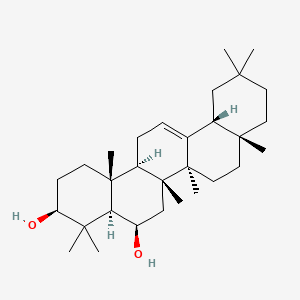 |
| parthenolide | 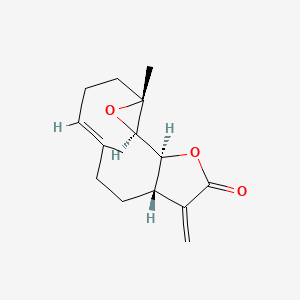 | gibberellin A98 | 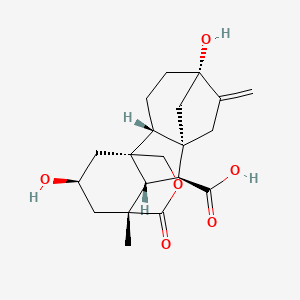 |
| xanthomicrol | 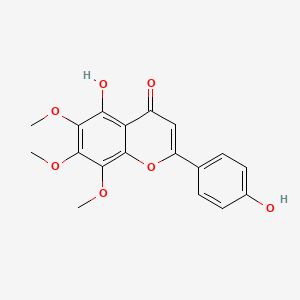 | "guaia-4,6-diene" | 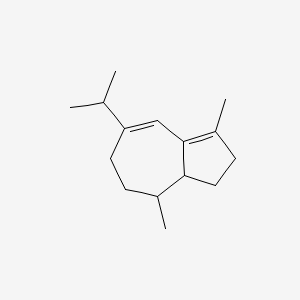 |
| euphornin L | 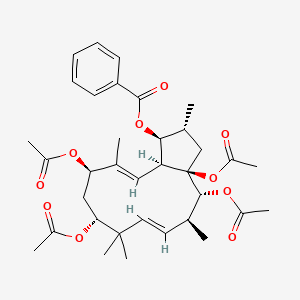 | multiorthoquinone | 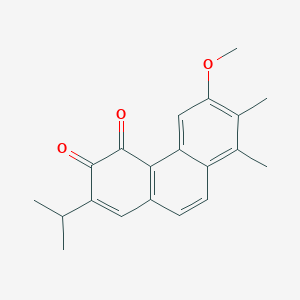 |
| arabinopyranoside | 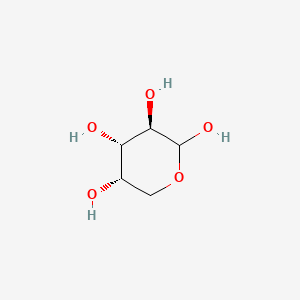 | β-acoradiene | 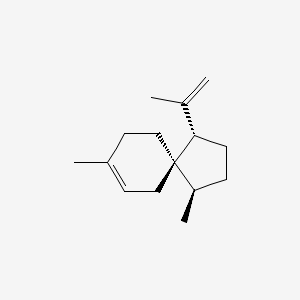 |
| (+)-pisatin | 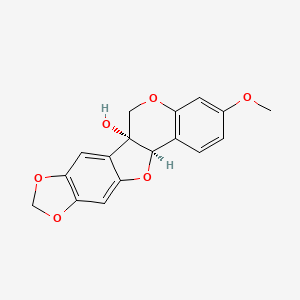 | 12-hydroxyjasmonic acid | 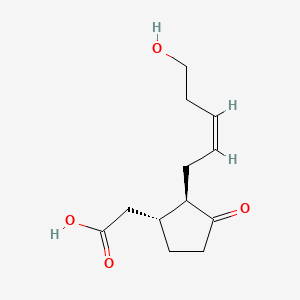 |
| (3E)-3-hexadecenoic acid | 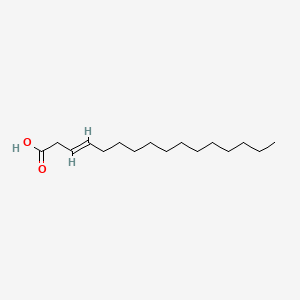 | asperuloside | 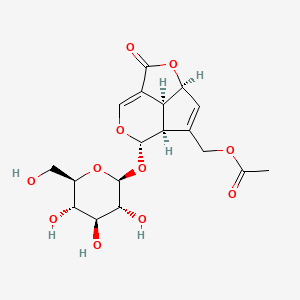 |
| 5α±-campestan-3-one | 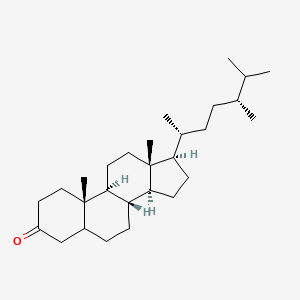 | kuwanone G | 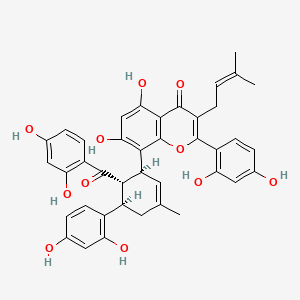 |
| cudraflavone B | 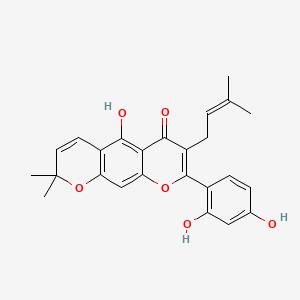 |  |  |

**Supplementary Table S4. Binding affinity output of targets with ligand molecules.**

| **Complex** | **Binding affinity (kj/mol)** | **RMSD** |
| --- | --- | --- |
| DPP4_Cudraflavone B | -10.9 | 0 |
| MAPK8_Liquiritin | -10.6 | 0 |
| MAPK8_Isoliquiritin | -10.6 | 0 |
| MAPK1_Hispaglabridin A | -9.9 | 0 |
| MIF_Cyclomorusin A | -9.7 | 0 |
| MAPK1_Cyclomulberrin | -9.4 | 0 |
| CTSB _Gibberellin A98 | -8.2 | 0 |
| TREM1_Multiorthoquinone | -7.5 | 0 |

**Supplementary Table S5.** The Names of targets and compounds obtained after MD analysis**.**

| **Target** | **Name** | **Classification** | **Source** |
| --- | --- | --- | --- |
| TREM1 | Multiorthoquinone | diterpenoid | Salvia multicaulis |
| MAPK8 | Liquiritin | flavanone glycoside | Glycyrrhiza glabra, Glycyrrhiza Uralensis |
| MAPK8 | Isoliquiritin | flavanone glycoside | Glycyrrhiza glabra |
| MAPK1 | Hispaglabridin A | hydroxyisoflavan | Glycyrrhiza glabra |
| CTSB | Gibberellin A98 | diterpenoid | Spinacea oleracea, Arabidopsis thaliana, Pisum sativum, Solanum lycopersicum, Zea mays |
| MAPK1 | Cyclomulberrin | flavonoid | Morus alba var. atropurpurea, Morus alba |
| MIF | Cyclomorusin A | flavonoid | Morus alba var. atropurpurea, Artocarpus altilis, Morus lhou, Ficus hirta, Morus alba, Morus australis, Morus bombycis, Morus nigra |
| DPP4 | Cudraflavone B | flavonoid | Cudrania tricuspidata, Artocarpus, Morus alba, Paulownia tomentosa |

**Supplementary Table S6.** Comparative plots of molecular dynamics**.**

| **Target Name** | **Compound Name** | **RMSD** | **Rg** | **Hbounds** | **RMSF** |
| --- | --- | --- | --- | --- | --- |
| TREM1 | Multiorthoquinone | 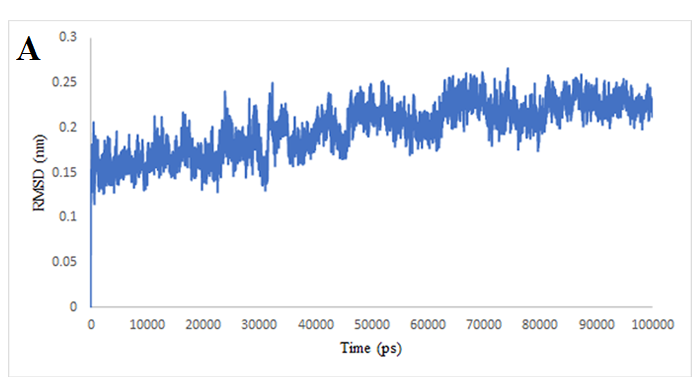 | 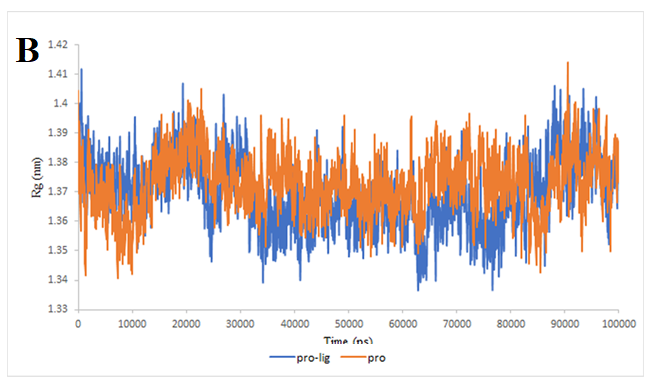 | 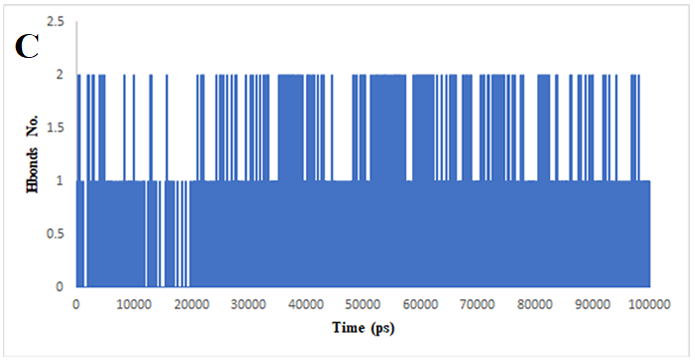 | 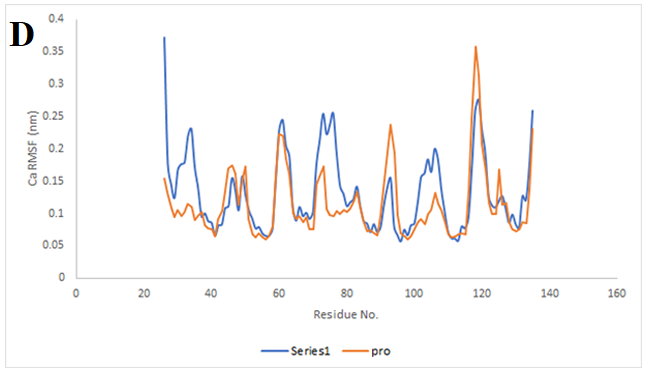 |
| MAPK8 | Liquiritin | 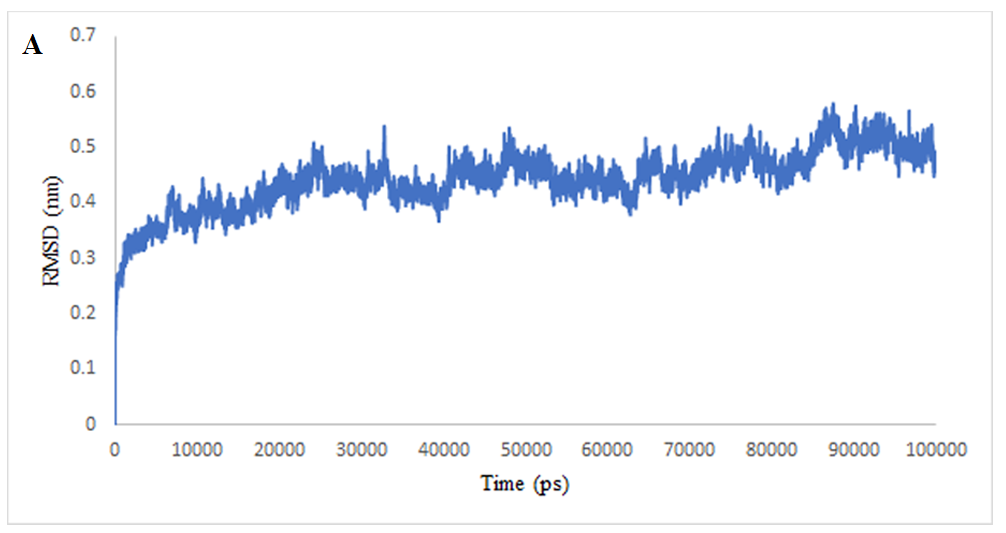 | 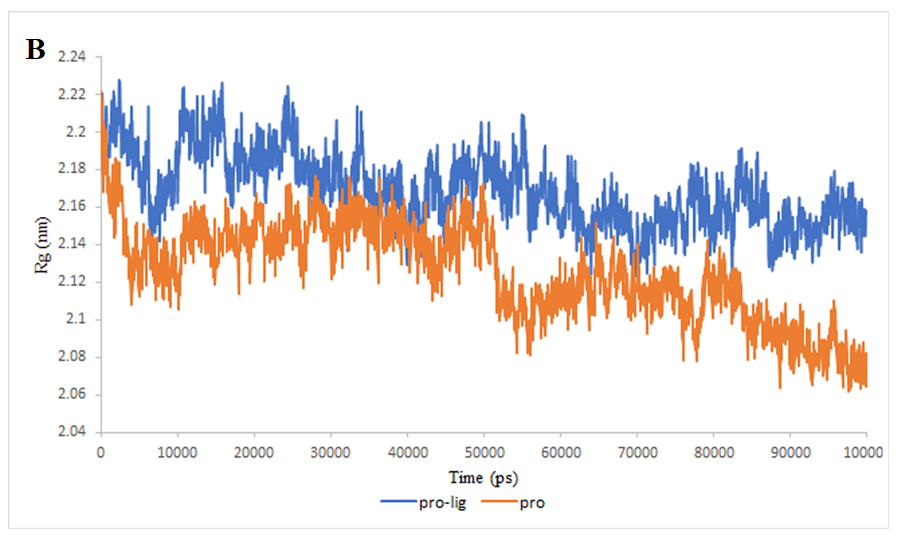 | 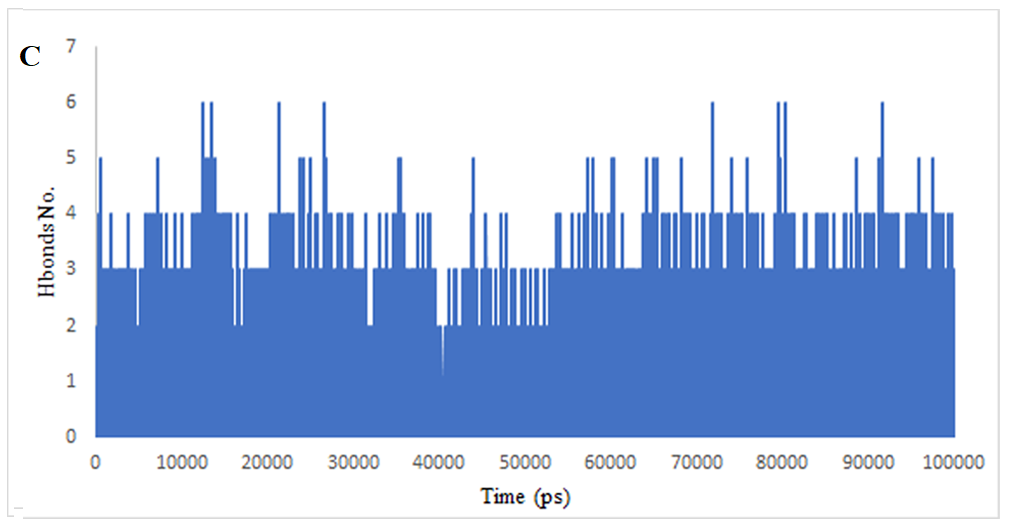 | 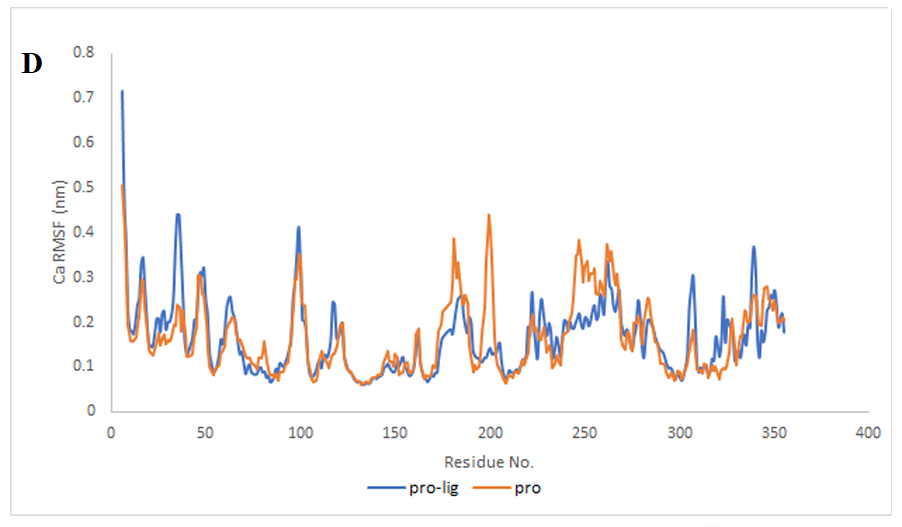 |
| MAPK8 | Isoliquiritin | 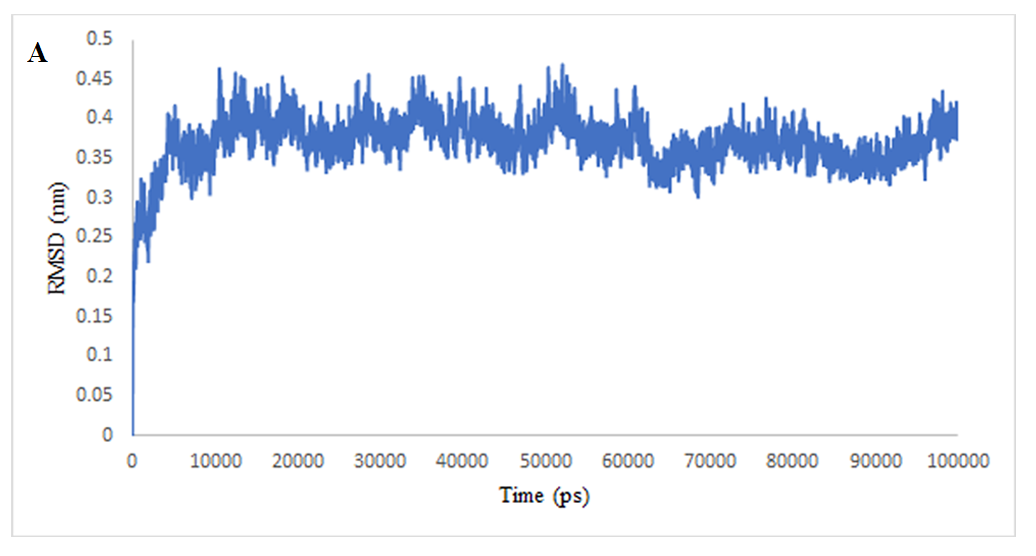 | 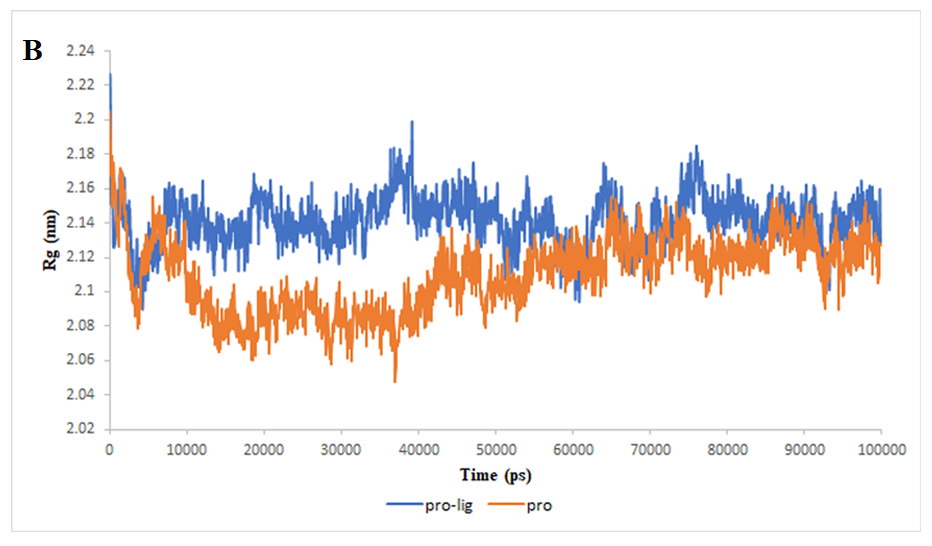 | 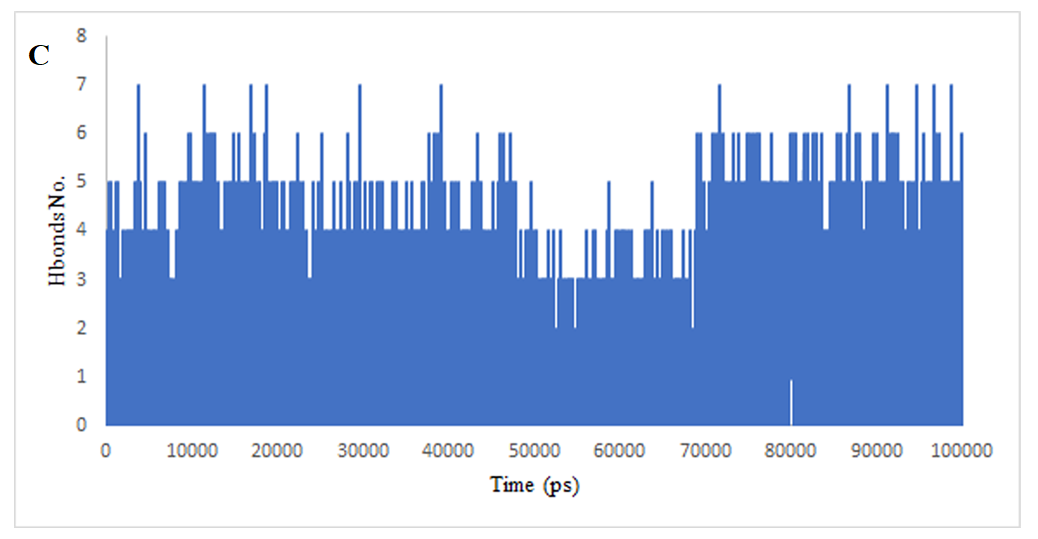 | 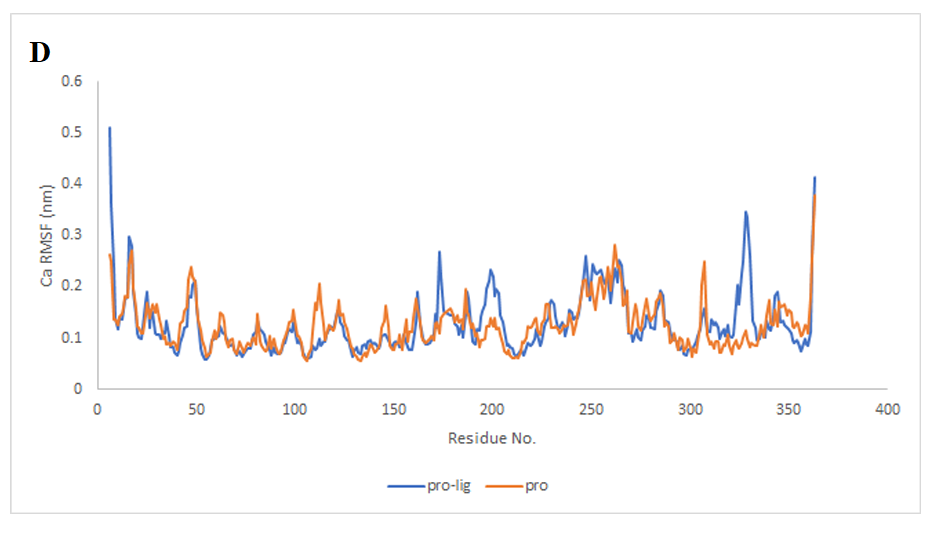 |
| MAPK1 | Hispaglabridin A | 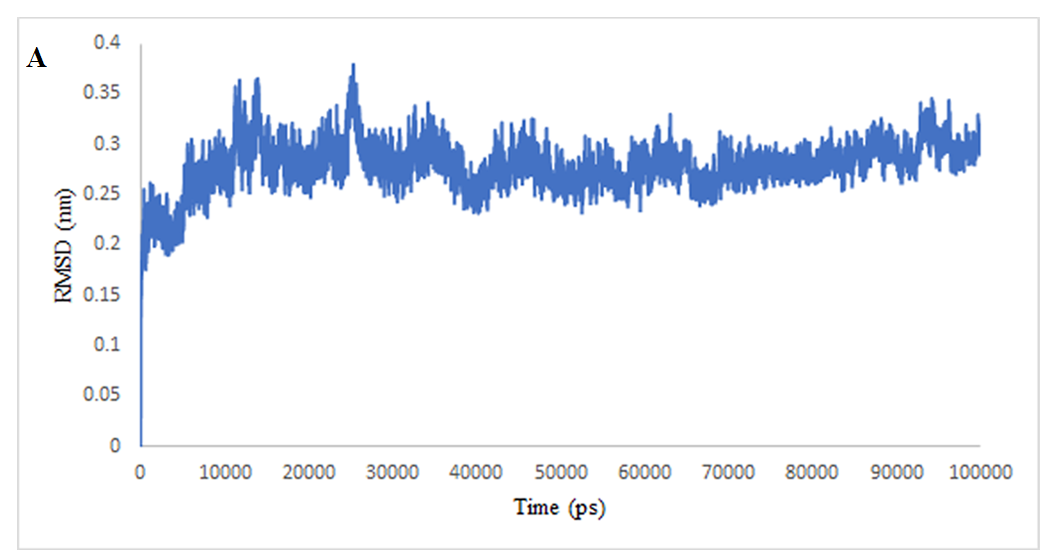 | 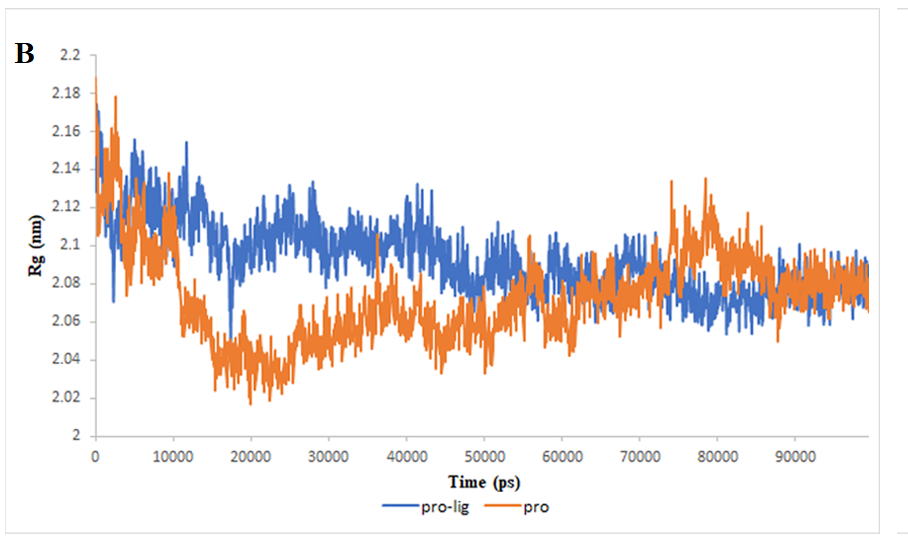 | 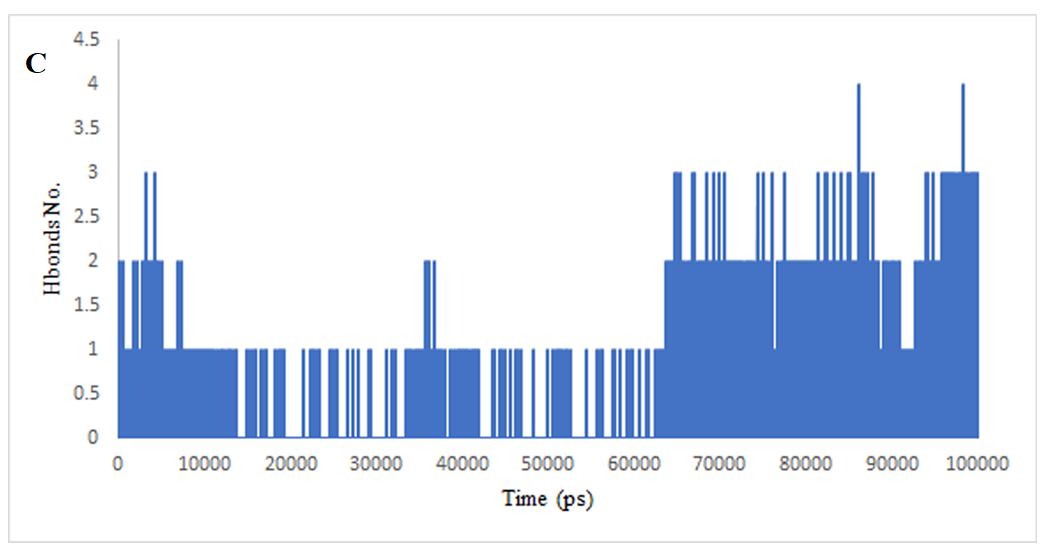 | 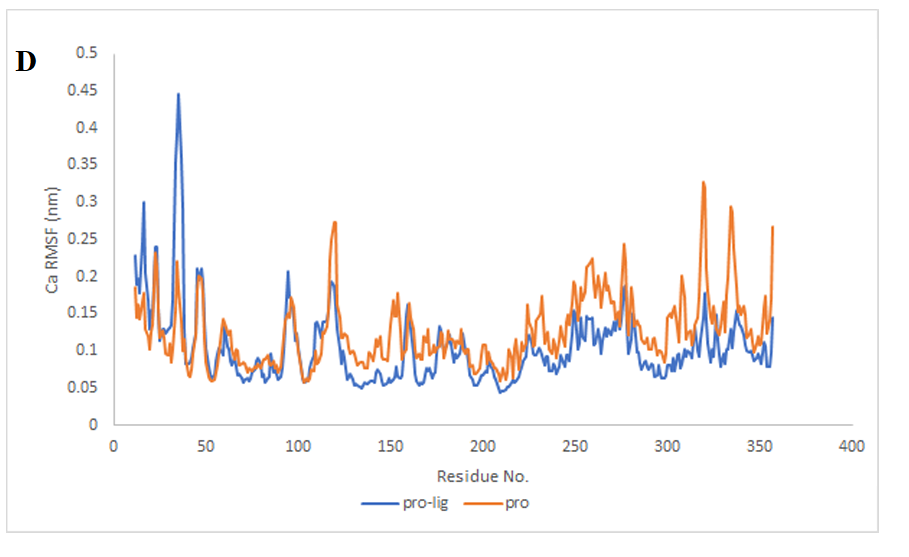 |
| MAPK1 | Cyclomulberrin | 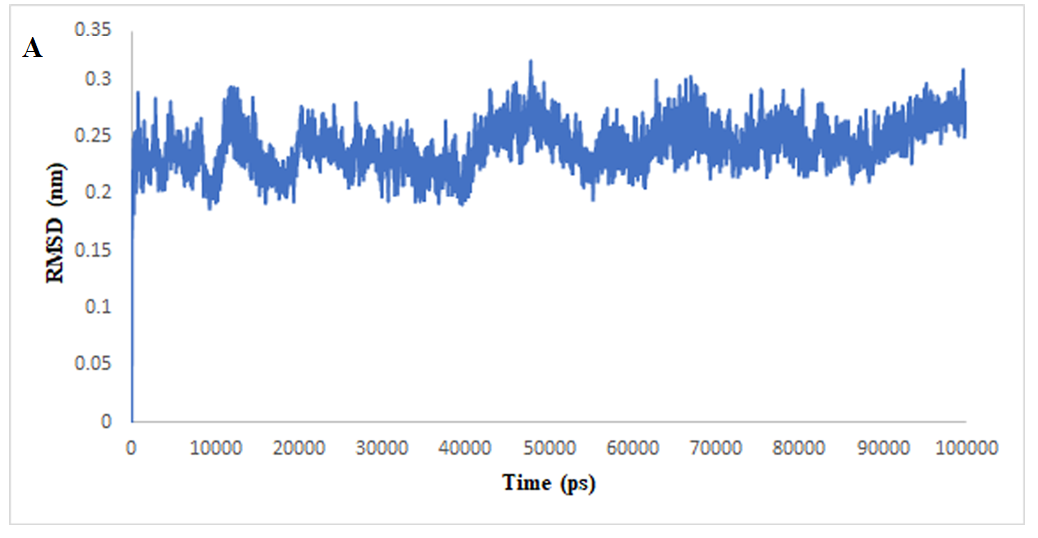 | 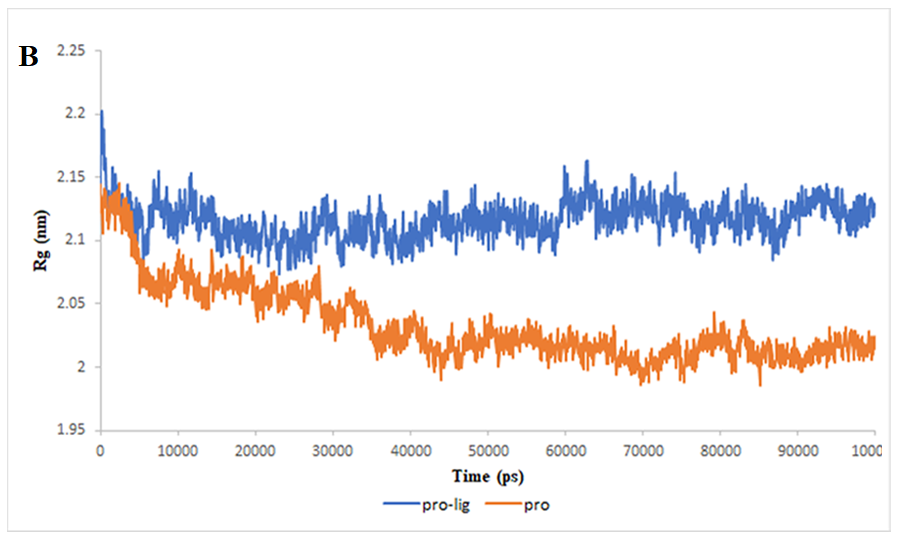 | 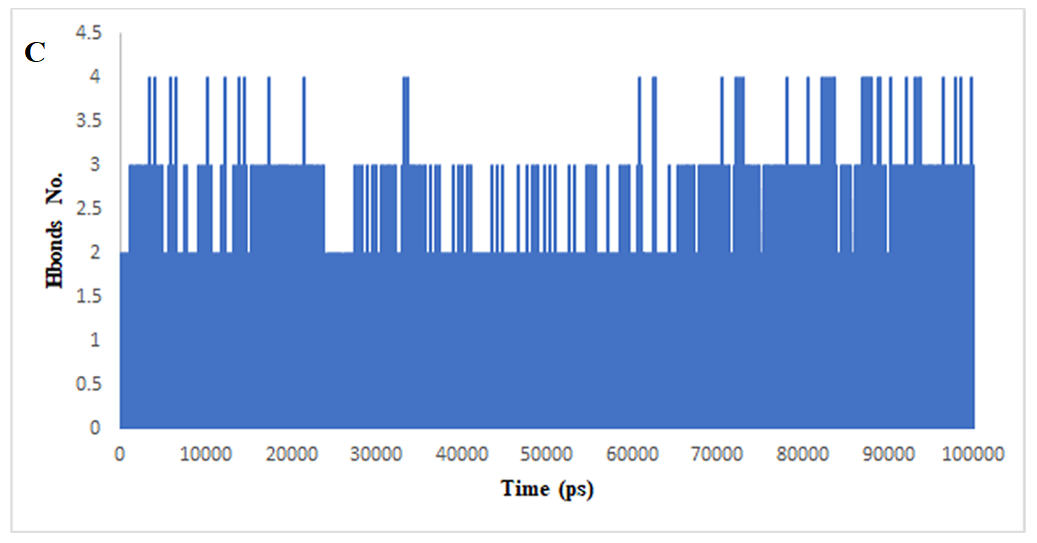 | 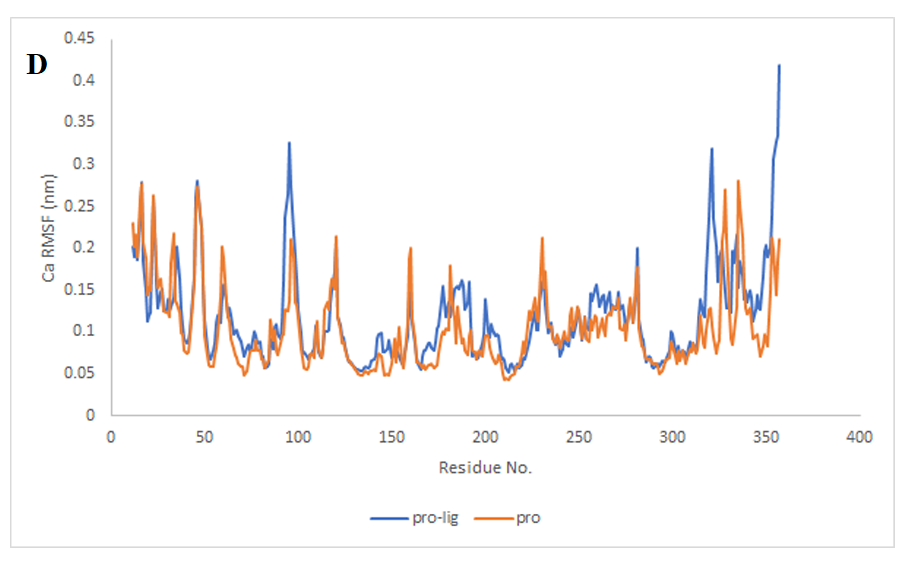 |
| DPP4 | Cudraflavone B | 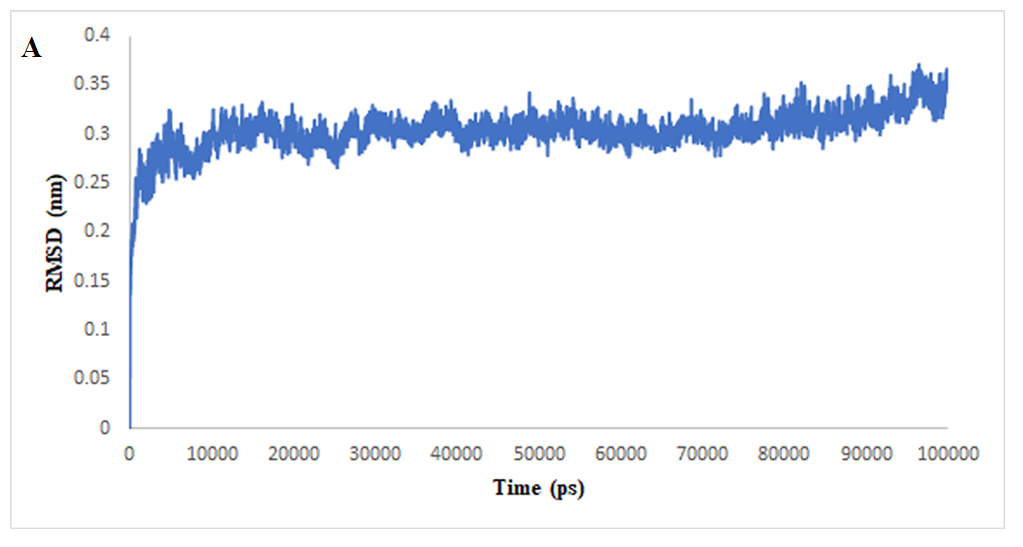 | 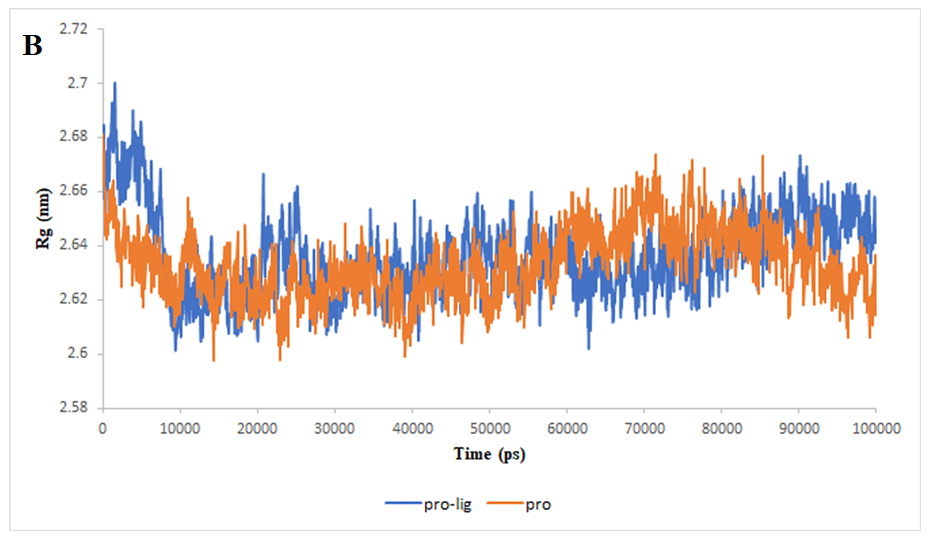 | 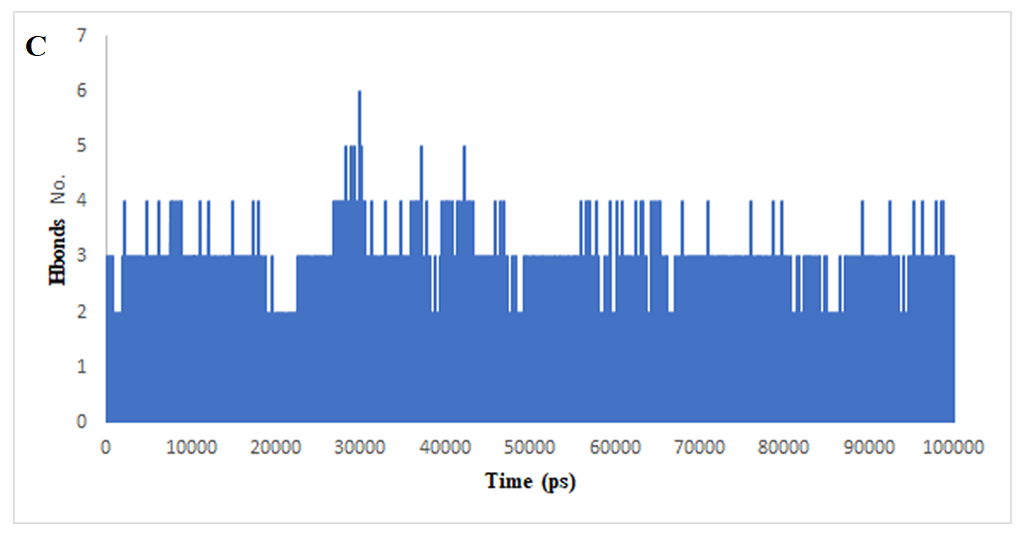 | 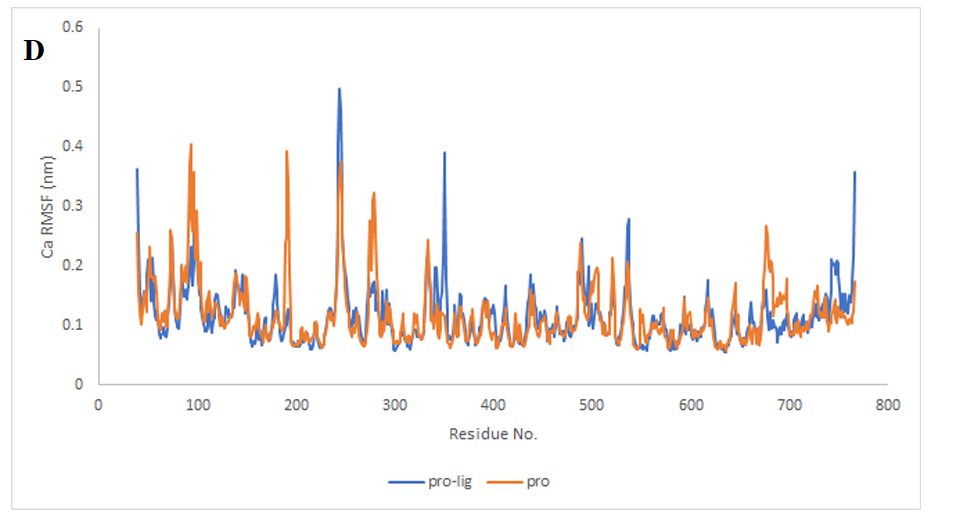 |
| CTSB | Gibberellin A98 | 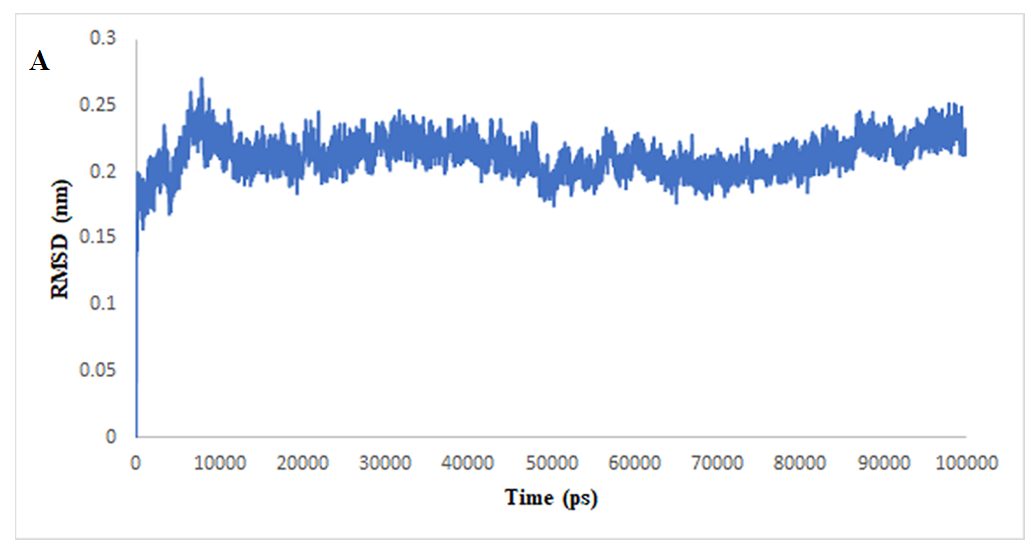 | 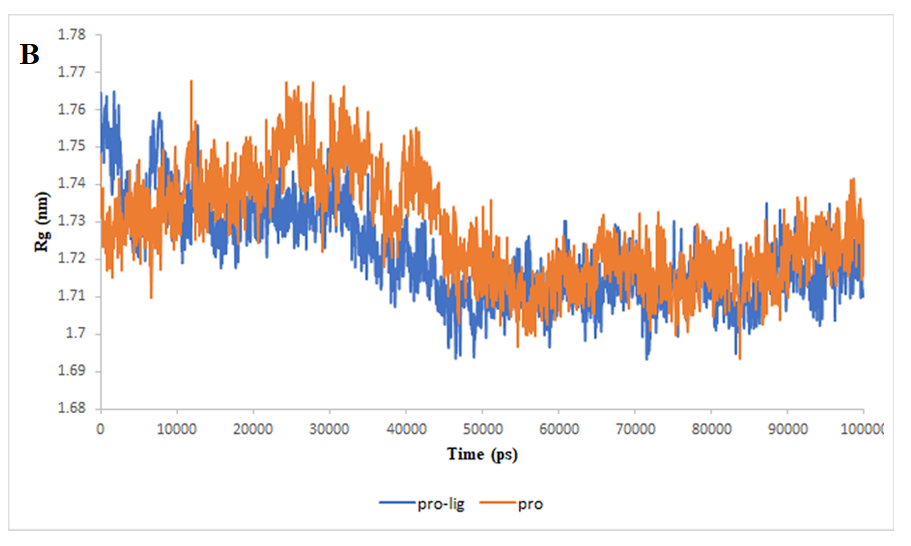 | 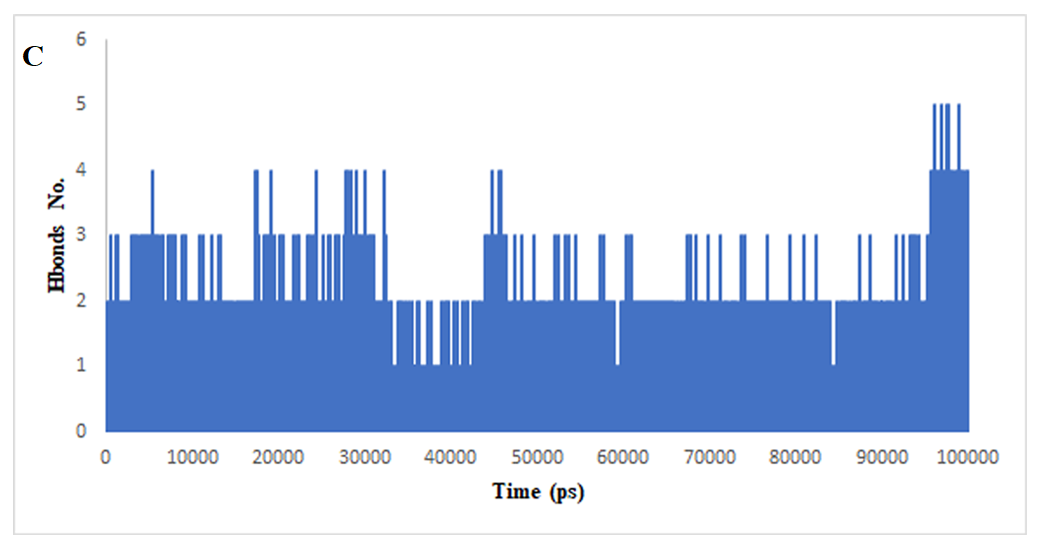 | 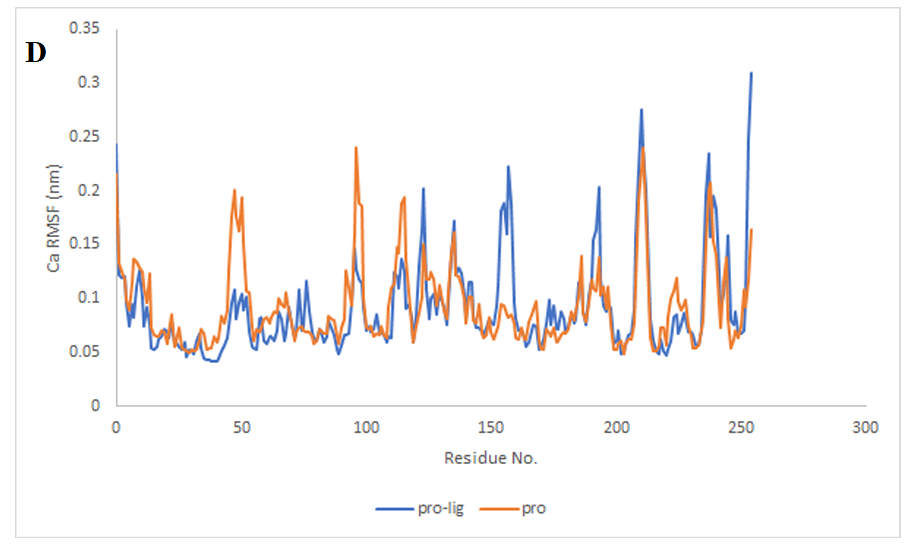 |
| MIF | Cyclomorusin A | 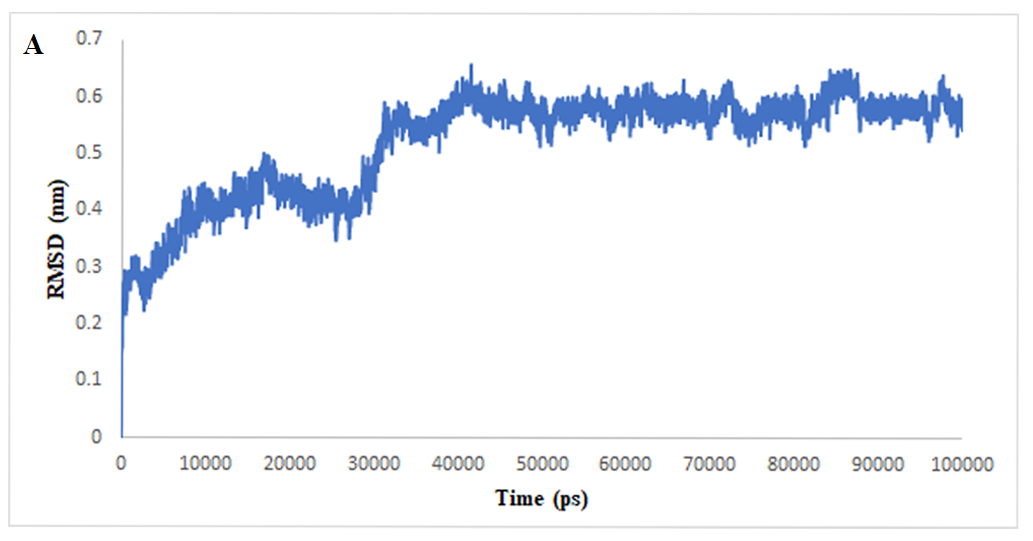 | 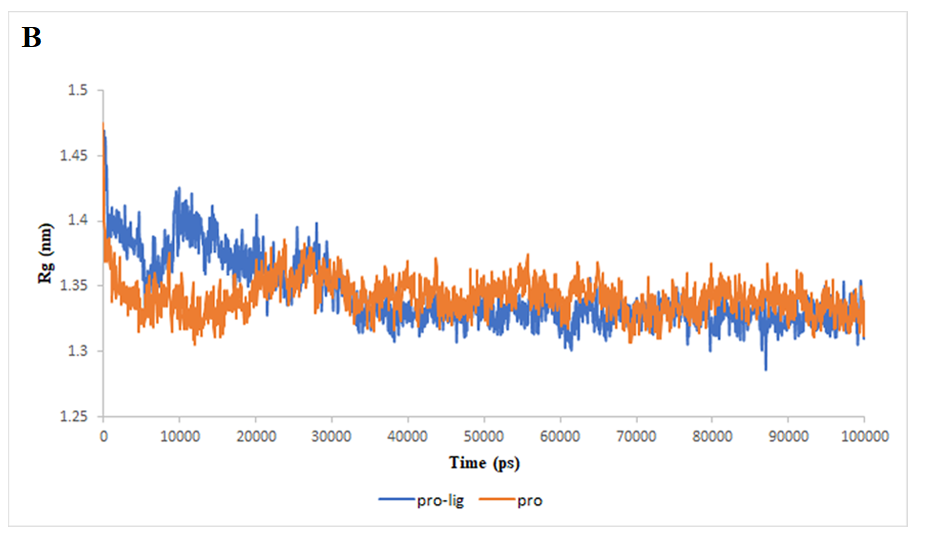 | 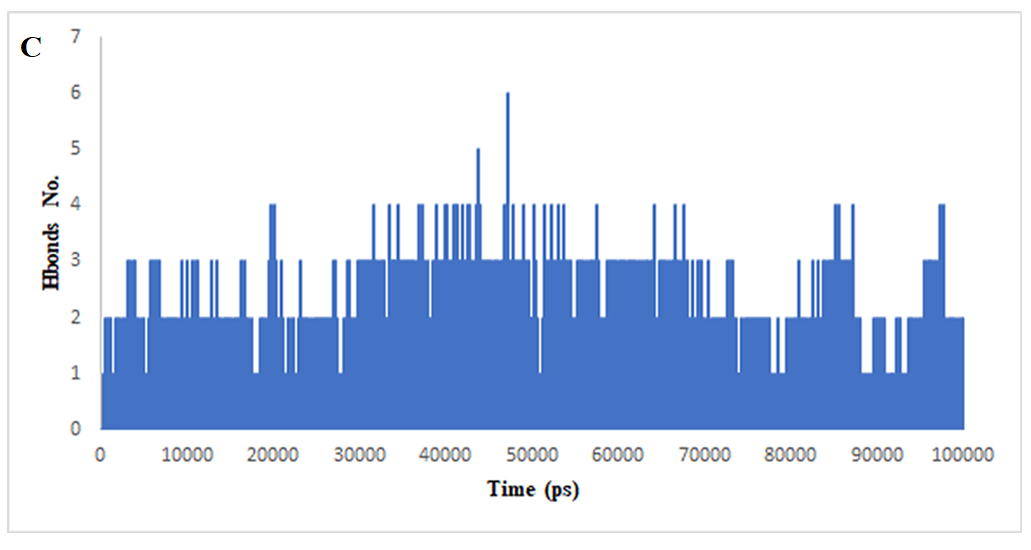 | 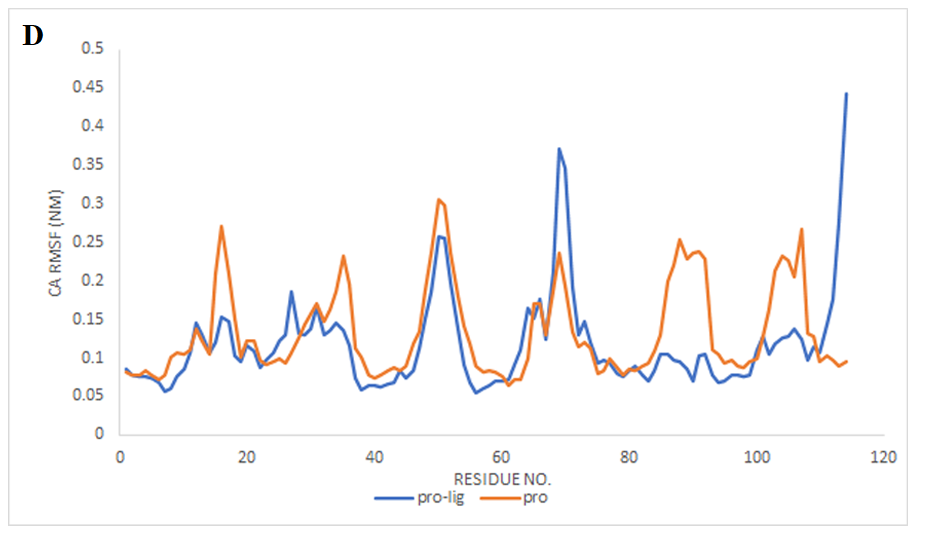 |

| **Target Name** | **Compound Name** | **A** | **B** | **C** |
| --- | --- | --- | --- | --- |
| TREM1 | Multiorthoquinone | 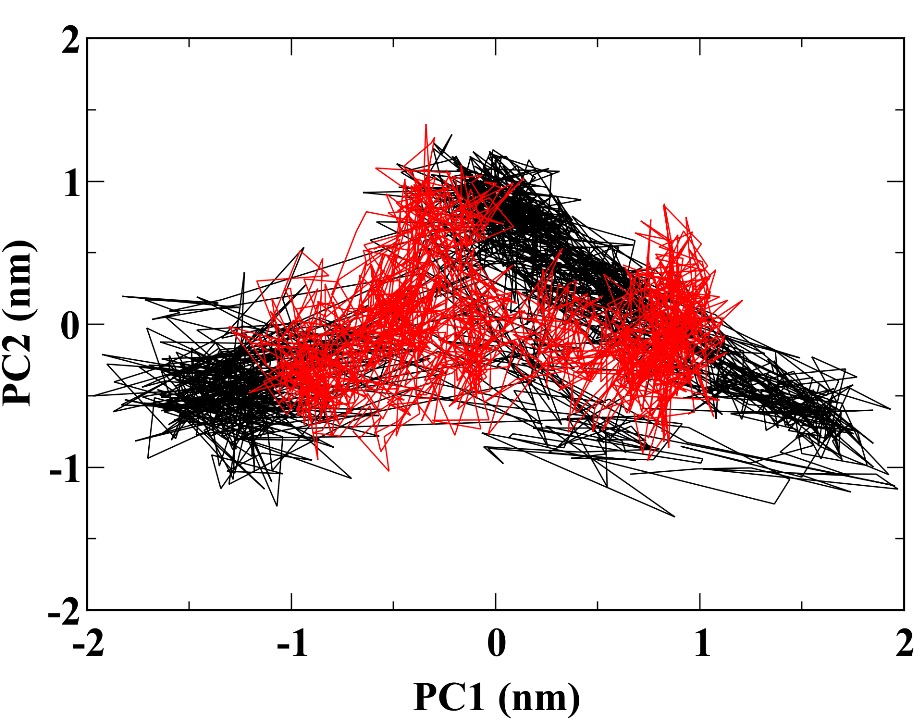 | 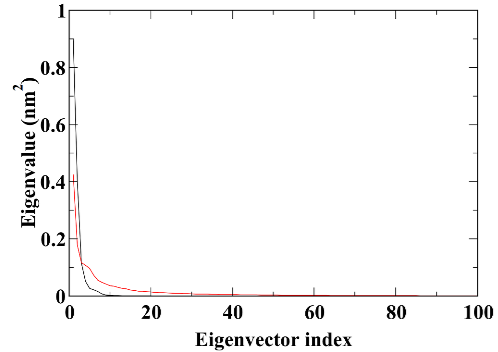 | 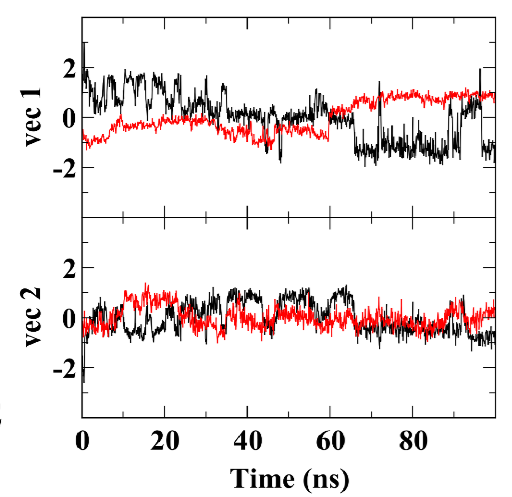 |
| MAPK8 | Liquiritin | 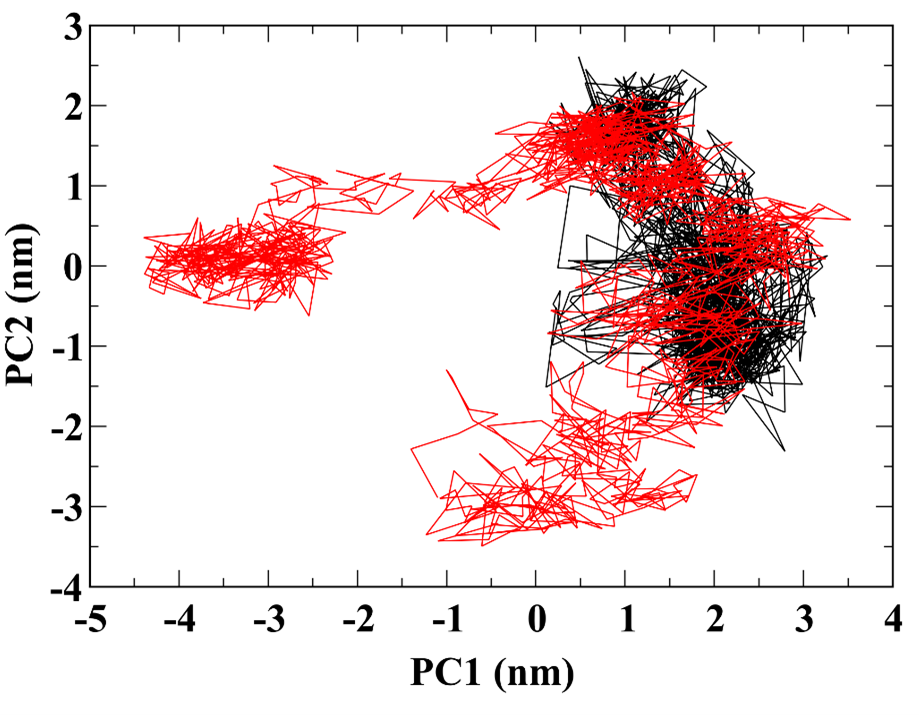 | 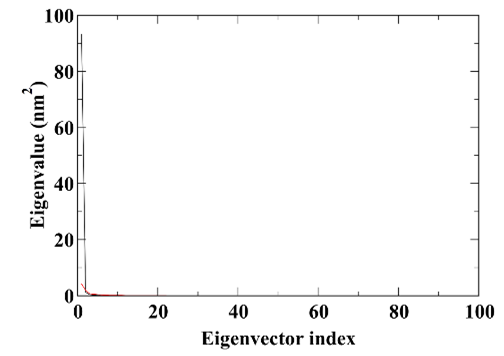 | 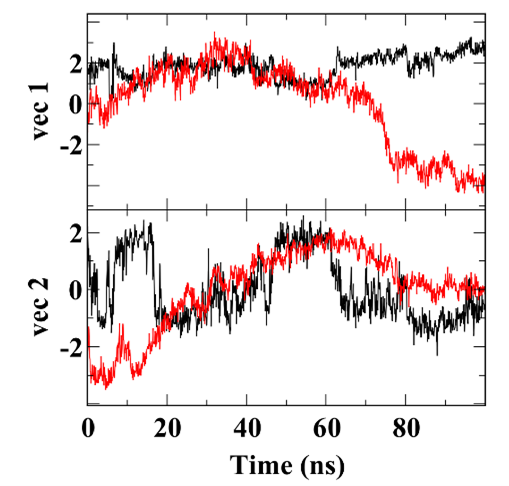 |
| MAPK8 | Isoliquiritin | 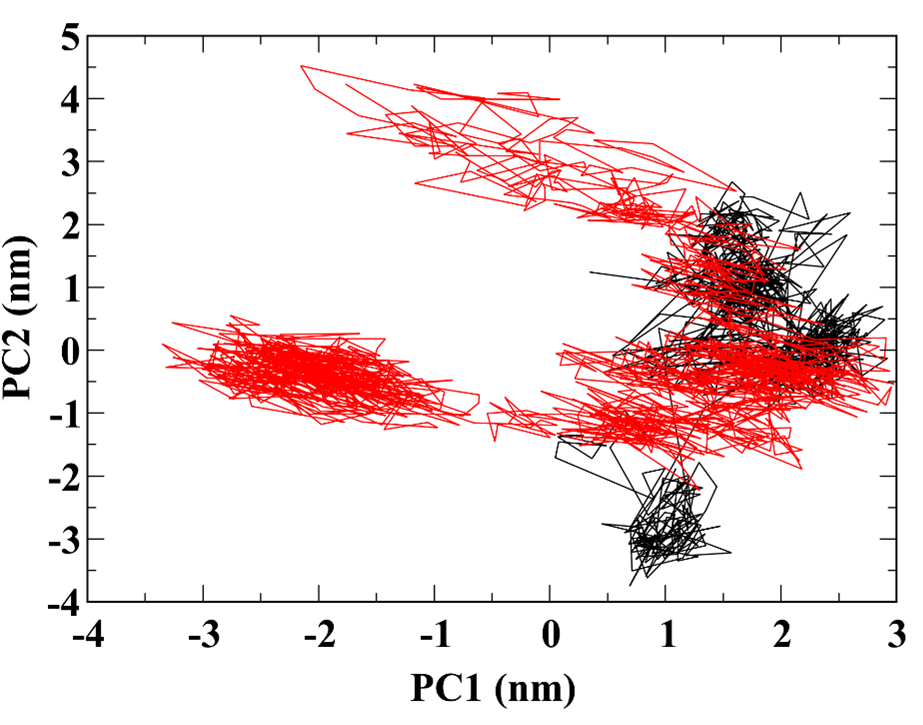 | 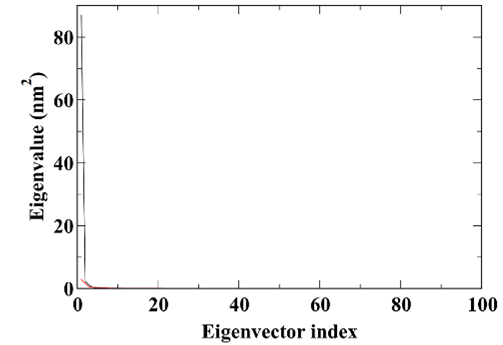 | 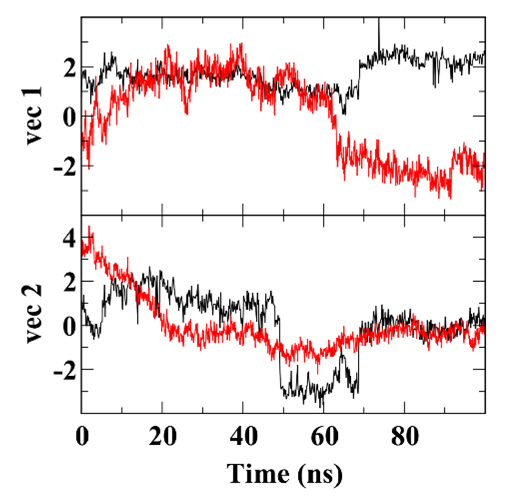 |
| MAPK1 | Hispaglabridin A | 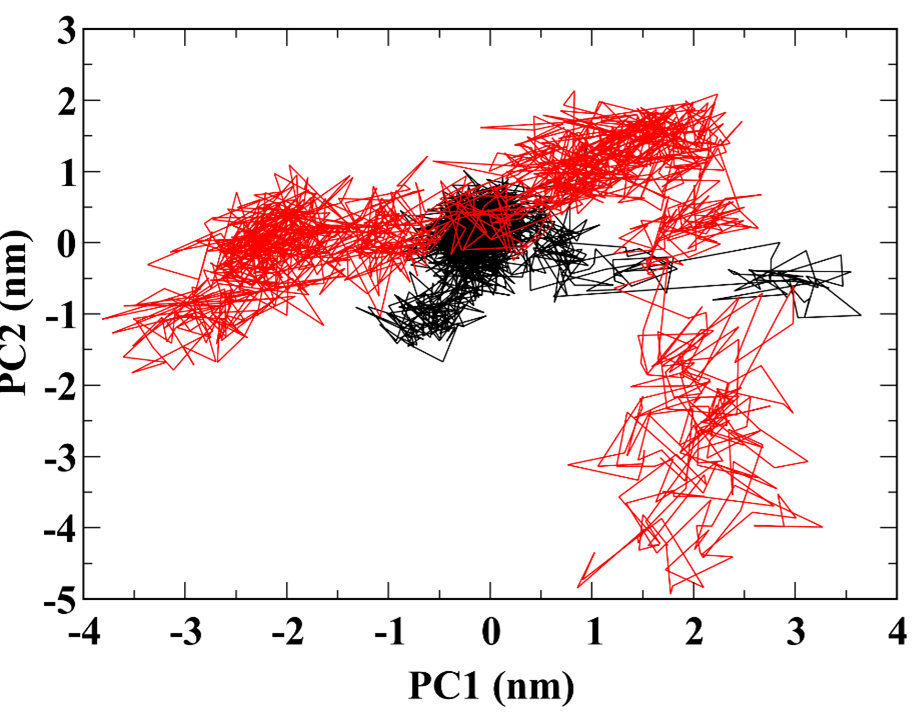 | 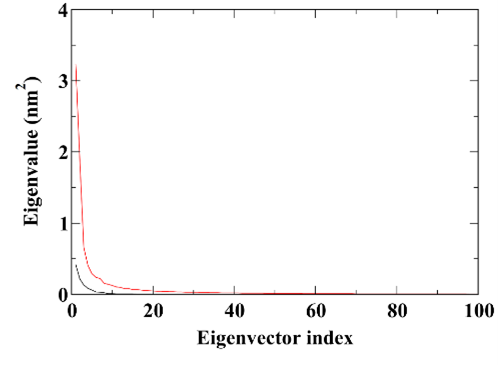 | 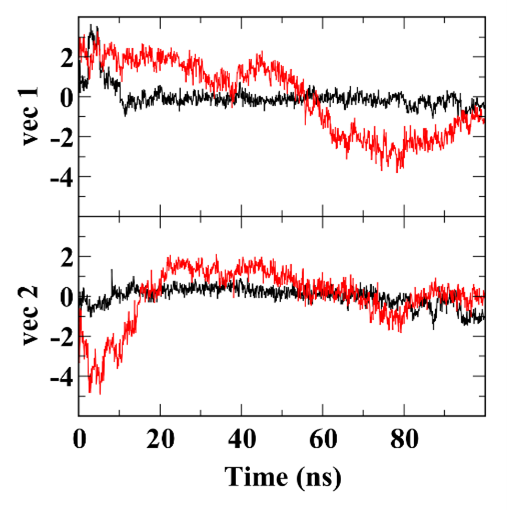 |
| MAPK1 | Cyclomulberrin | 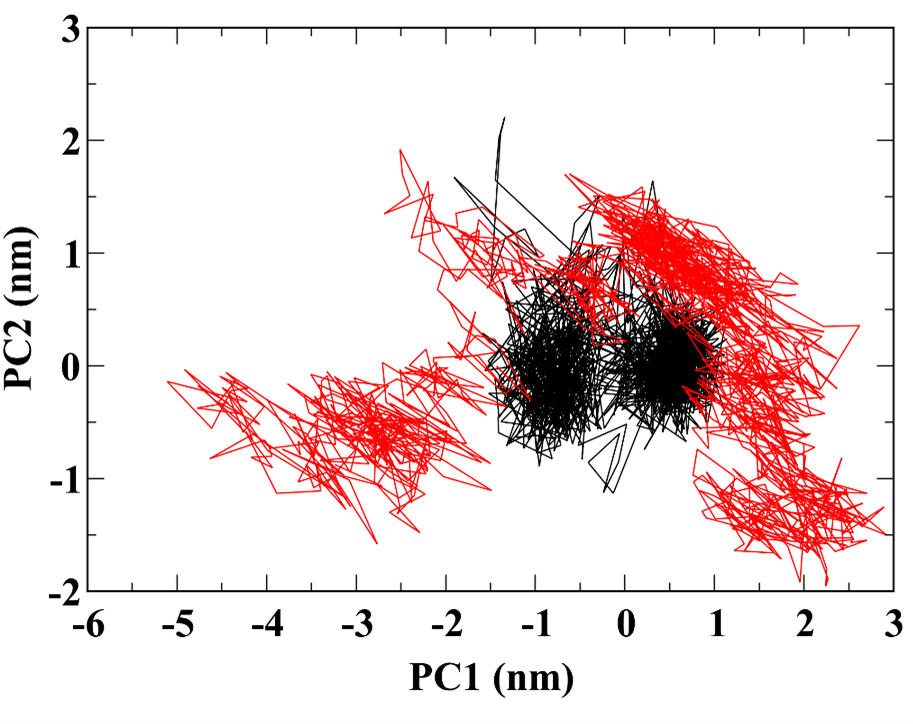 | 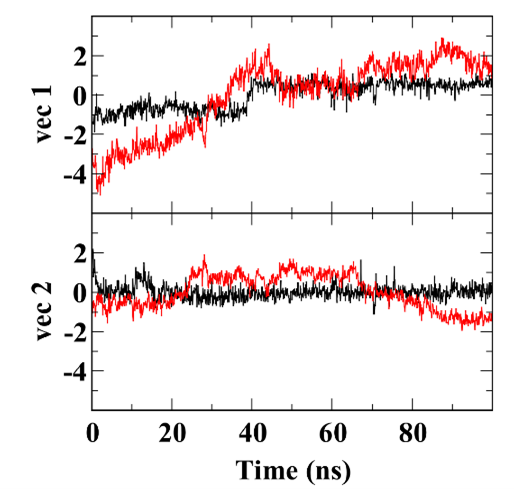 | 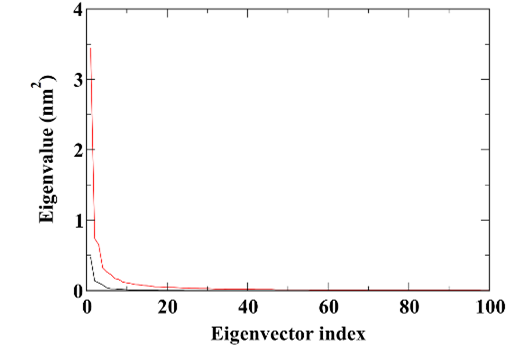 |
| DPP4 | Cudraflavone B | 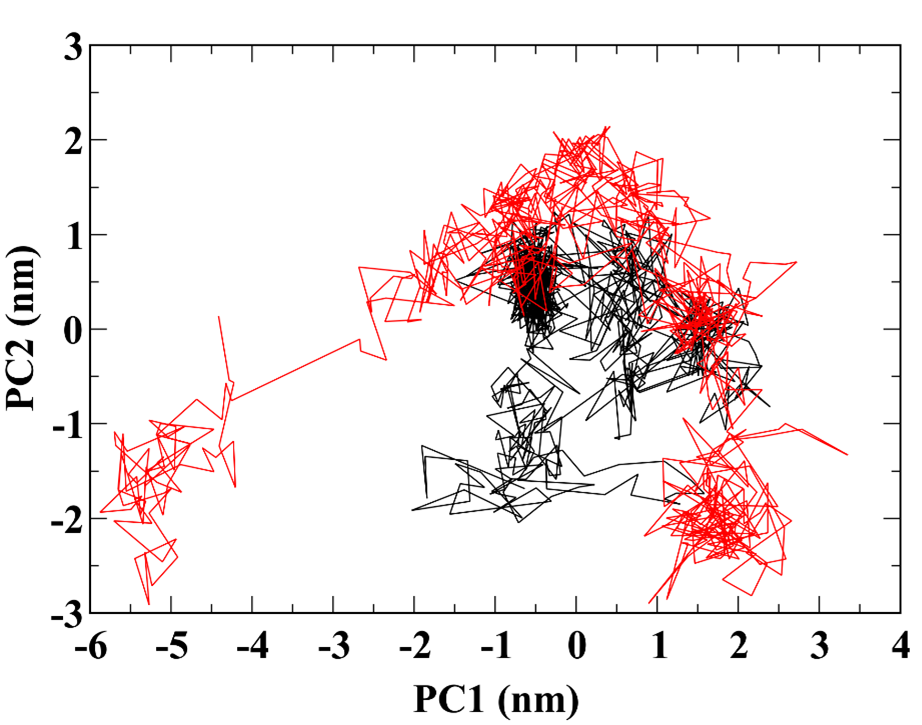 | 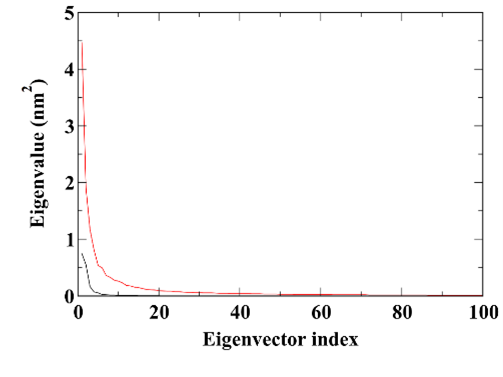 | 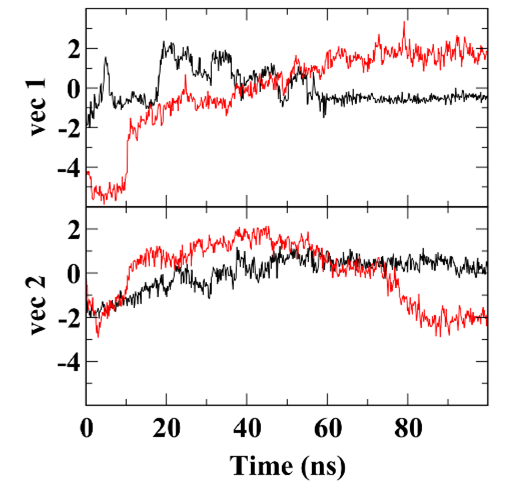 |
| CTSB | Gibberellin A98 | 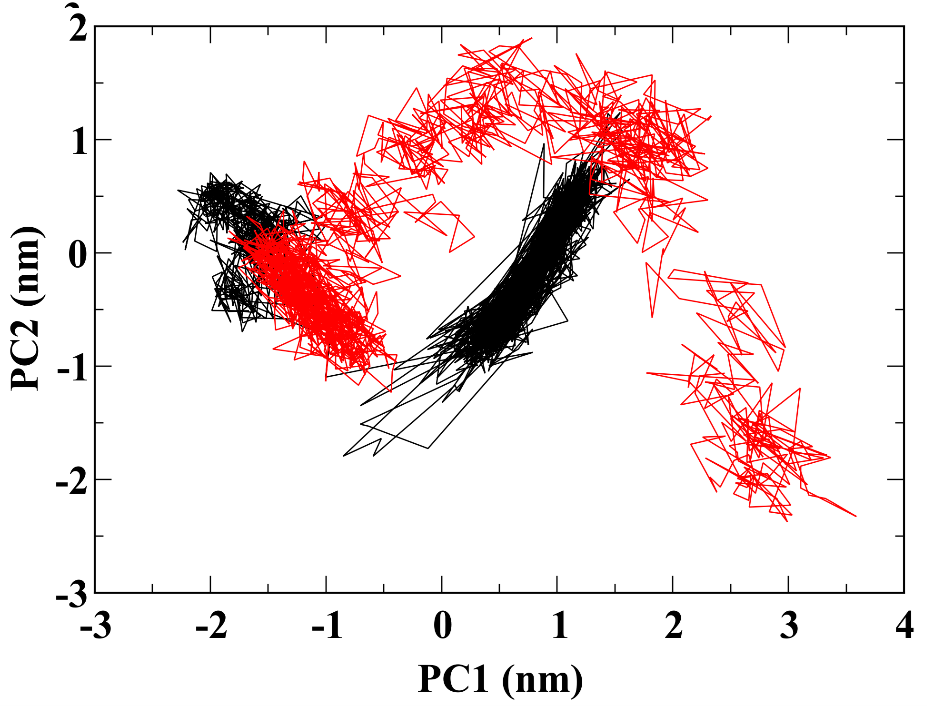 | 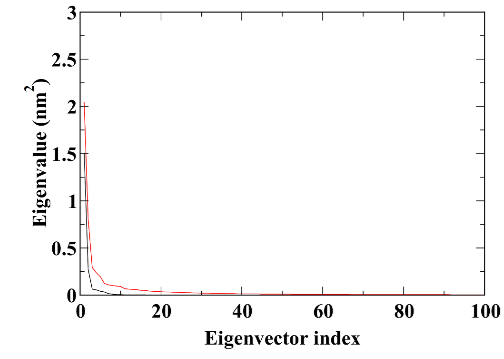 | 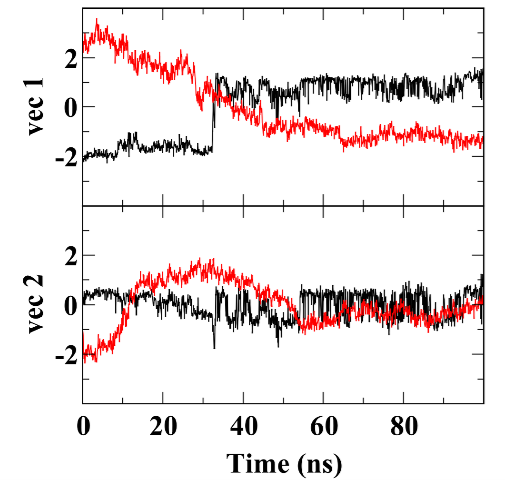 |
| MIF | Cyclomorusin A | 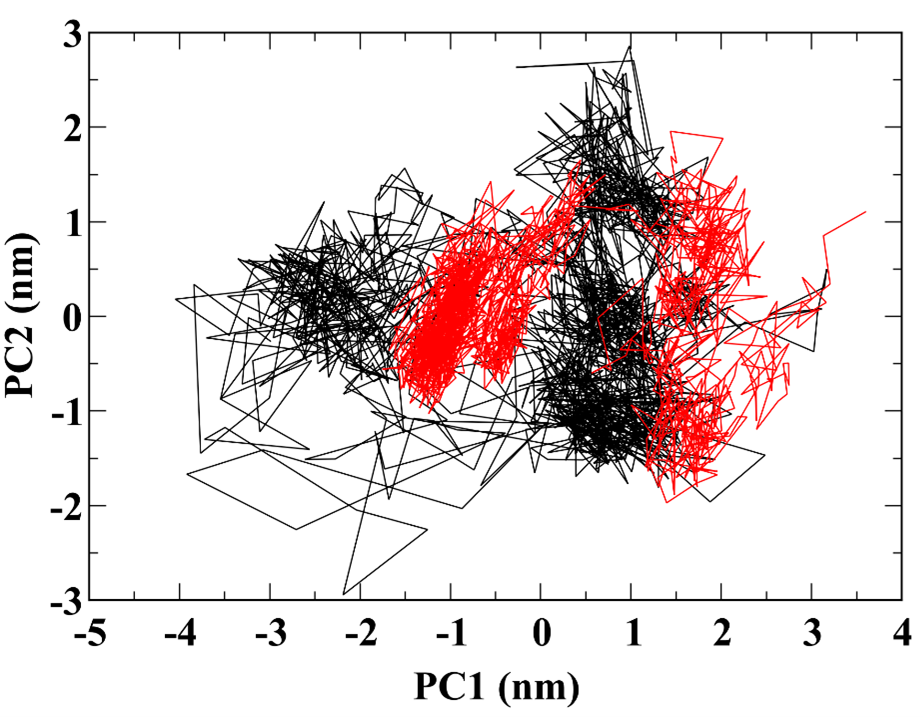 | 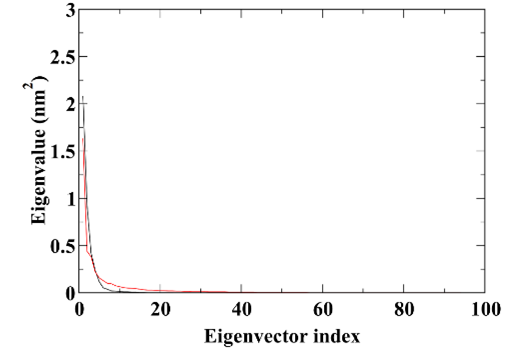 | 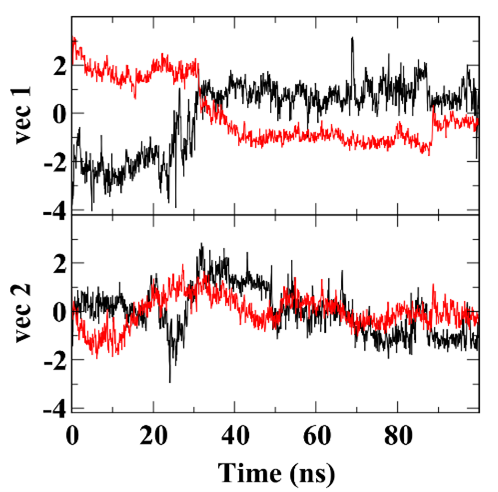 |

**Supplementary Figure S1**. Principal component analysis (PCA) of proteins and protein-ligand complexes: (A) Collective motion using MD trajectory projections on the two eigenvectors corresponding to the ﬁrst two PCs (PC2 vs. PC1), (B) eigenvectors of the covariance matrix, (C) projection of each eigenvector during the simulation time. unbound proteins are shown in red, protein-ligand complexes in black
